# Rab35 and glucocorticoids regulate APP and BACE1 trafficking to modulate Aβ production

**DOI:** 10.1038/s41419-021-04433-w

**Published:** 2021-12-08

**Authors:** Viktoriya Zhuravleva, João Vaz-Silva, Mei Zhu, Patricia Gomes, Joana M. Silva, Nuno Sousa, Ioannis Sotiropoulos, Clarissa L. Waites

**Affiliations:** 1 Neurobiology and Behavior Graduate Program, Columbia University, New York, NY 10032 USA; 2 Life and Health Sciences Research Institute (ICVS), School of Medicine, University of Minho, Campus de Gualtar, 4710-057 Braga, Portugal; 3 ICVS/3B’s - PT Government Associate Laboratory, 4710-057 Braga/Guimarães, Portugal; 4 Department of Pathology and Cell Biology, Taub Institute for Research on Alzheimer’s Disease and Aging Brain, Columbia University Irving Medical Center, New York, NY 10032 USA

**Keywords:** Cellular neuroscience, Alzheimer's disease

## Abstract

Chronic stress and elevated glucocorticoids (GCs), the major stress hormones, are risk factors for Alzheimer’s disease (AD) and promote AD pathomechanisms, including overproduction of toxic amyloid-β (Aβ) peptides and intraneuronal accumulation of hyperphosphorylated Tau protein. The latter is linked to downregulation of the small GTPase Rab35, which mediates Tau degradation via the endolysosomal pathway. Whether Rab35 is also involved in Aβ overproduction remains an open question. Here, we find that hippocampal Rab35 levels are decreased not only by stress/GC but also by aging, another AD risk factor. Moreover, we show that Rab35 negatively regulates Aβ production by sorting amyloid precursor protein (APP) and β-secretase (BACE1) out of the endosomal network, where they interact to produce Aβ. Interestingly, Rab35 coordinates distinct intracellular trafficking steps for BACE1 and APP, mediated by its effectors OCRL and ACAP2, respectively. Finally, we demonstrate that Rab35 overexpression prevents the amyloidogenic trafficking of APP and BACE1 induced by high GC levels. These studies identify Rab35 as a key regulator of APP processing and suggest that its downregulation may contribute to stress-related and AD-related amyloidogenesis.

## Introduction

Alzheimer’s disease (AD) is the most common neurodegenerative disease and cause of dementia. While aging constitutes the highest risk factor for AD [1], emerging evidence indicates that chronic stress and high circulating levels of glucocorticoids (GCs), the main stress hormones, are also risk factors for the disease [2–4]. Indeed, recent work has shown that chronic unpredictable stress and high GC levels induce AD-like pathology in animal models, including the overproduction of toxic amyloid-beta (Aβ) peptides, as well as the accumulation, hyperphosphorylation, and synaptic missorting of Tau protein, leading to synaptotoxicity and memory impairment [4–9].

Our previous work showed that GC-induced Tau pathology is precipitated in part by transcriptional downregulation of the small GTPase Rab35 [10], a master regulator of endosomal trafficking. In particular, we found that Rab35 promotes Tau degradation via the endolysosomal pathway, and that its downregulation by GCs leads to intraneuronal Tau accumulation [10]. AAV-mediated overexpression of Rab35 in the rodent hippocampus was sufficient to prevent GC-induced Tau accumulation and downstream dendrite and spine loss [10]. These findings demonstrate that GCs precipitate Tau pathology by disrupting Rab35-mediated endolysosomal trafficking. Given that Rab35 mediates multiple other intracellular trafficking events, including the retrograde trafficking of mannose-6-phosphate receptors [11] and the recycling of cell-surface receptors and adhesion molecules [12–14], its GC-mediated downregulation could impact amyloid precursor protein (APP) trafficking and contribute to stress-induced Aβ production.

Trafficking of APP and β-secretase1 (BACE1) into the endosomal network is an essential step in Aβ production [15]. BACE1 mediates the rate-limiting cleavage step in amyloidogenic processing of APP into Aβ, and endosomes are major sites of Aβ generation due to their optimal acidic pH for BACE1 activity [15, 16]. Intriguingly, many of the recently identified genetic risk factors for late-onset AD are linked to endosomal protein trafficking and have been shown to induce endosomal dysfunction and prolong the residence times of APP and/or BACE1 in endosomes [17, 18]. The ~60 member family of Rab GTPases are key regulators of endosomal protein trafficking and have been implicated in the etiology of AD [19, 20]. Indeed, a subset of Rabs, including Rab35, were identified as regulators of Aβ production in a loss-of-function screen of Rab GTPases performed in non-neuronal cells [19]. However, it is yet unclear whether Rab35 regulates APP or BACE1 trafficking and Aβ production in neurons, and if so, how this function is impacted by stress/GCs.

Here, we demonstrate that Rab35 levels decrease in the hippocampus in response to chronic stress and aging, both AD risk factors, suggesting that Rab35 reduction may be a precipitating factor for AD-related pathomechanisms. Consistent with these observations, we show that Rab35 inhibits Aβ production by stimulating APP and BACE1 trafficking out of the endosomal network. Interestingly, Rab35 regulates distinct trafficking steps for APP and BACE1, mediated by different effectors (ACAP2 and OCRL, respectively). Finally, we find that high GC levels alter the endosomal trafficking of APP and BACE1 to increase their interaction, and that Rab35 overexpression counteracts these effects. Together, our findings implicate Rab35 as a negative regulator of amyloidogenic APP processing and suggest that its downregulation contributes to Aβ production during aging or after prolonged exposure to stressful conditions.

## Materials and methods

### Primary neurons and cell lines

Primary neuronal cultures were prepared from E18 Sprague Dawley rat embryos and maintained for 14 DIV before use, as described previously [21]. Neuro2a (N2a) neuroblastoma cells (ATCC CCL-131) were grown in DMEM-GlutaMAX (ThermoFisher) with 10% FBS (Atlanta Biological) and Anti-Anti (ThermoFisher) and kept at 37 °C in 5% CO_2_. During dexamethasone treatment in N2a cells, FBS in the growth media was reduced to 3%. Human iPSC-derived neuronal primary cultures were differentiated by dual inhibition of SMAD signaling, as previously described [22–24]. Sample sizes for neurons and N2a cells were chosen by approximation, assuming that effect sizes would be similar to those in Vaz-Silva et al. [10] and estimating that 20 or more cells are required to detect differences between groups.

### Pharmacological treatments

Pharmacological agents were used in the following concentrations and time courses: cycloheximide (Calbiochem, 0.2 µg/µl, 2, 4, or 8 h), dexamethasone (Invivogen, 10 µM, 24 h).

### Lentivirus production, transduction, and DNA transfection

With the exception of APP:VN and BACE:VC constructs (a gift from Dr. Subhojit Roy, University of California, San Diego, USA), DNA constructs used here were described previously [10, 21]. Lentivirus was also produced as described previously [21]. Neurons were transduced with 50–150 µl of lentiviral supernatant per well (12-well plates) or 10–40 µl per coverslip (24-well plates) either at three DIV for shRNA transduction or ten DIV for overexpression experiments. Respective controls were transduced on the same day for all experimental conditions. Primary neuronal cultures were collected for immunoblotting or immunocytochemistry at 14 DIV. N2a cells were transfected using Lipofectamine 3000 approximately 24 h after plating, according to the manufacturer’s instructions. For the bimolecular fluorescence complementation assay, double transfection of APP:VN and BACE1:VC constructs was performed 48 h after transfection with Rab35, to allow for longer expression of the construct. Cells were then fixed and analyzed 18 h after the APP:VN and BACE1:VC transfection.

### Proximity ligation assay (PLA)

PLA was performed in primary hippocampal neurons at 37 °C in a humidity chamber, according to the manufacturer’s instructions (Duolink, Sigma). The primary antibody pairs used were C1/6.1 (anti-APP, Mouse; Biolegend) and anti-BACE1 (Rabbit, Cell Signaling Technology). Coverslips were mounted using Duolink In situ Mounting Media with DAPI.

### Aβ measurements

Human iPSC-derived neuronal cultures were kept for 3 days post-transduction, after which 50% of the media was changed. Conditioned media was collected after 72 h, centrifuged at 2000 rcf for 5 min, and stored at −80 °C. Aβ42 and Aβ40 levels were measured using V-PLEX Aβ Peptide Panel 1 (4G8) Kit (MesoScaleDiscovery, MSD) following the manufacturer’s protocol.

### Animal experiments

All animals used in this study were maintained under standard laboratory conditions in accordance with the guidelines for the care and handling of laboratory animals, described in the Directive 2010/63/EU. All experiments were approved by the animal ethics committee of the University of Minho and the Portuguese national authority for animal experimentation (DGV9457). For chronic unpredictable stress (CUS) and Aβ infusion experiments, 12-month-old male Wistar rats (Charles River Laboratories, France) were divided into three groups: control, CUS, and Aβ-infused (*N* = 8–9 per group). The 4 week CUS paradigm was performed as previously described [5]. Control animals remained in their home cages during this period. At the end of the CUS paradigm, all animals were implanted with Alzet miniosmotic pumps (DURECT, 2002 model) for i.c.v. delivery of Aβ1-40 (Eurogentec; 25 μg/200 μl, 0.5 μl per hour) or saline for 14 days. For Aβ1–40 or saline infusion, cannulae (Alzet Brain Infusion Kit) were implanted in the left lateral ventricle using the following coordinates from Bregma: −0.6 mm anteroposterior, −1.4 mm mediolateral, −3.5 mm dorsoventral according to Paxinos and Watson [25]. Pump and cannula implantation were done under anesthesia [75 mg/kg ketamine (Imalgene, Merial) and 1 mg/kg medatomidine (Dorbene, Cymedica)]. ﻿For the aged rat study, young (4 month-old) and aged (22–24 month-old) male Wistar rats were used (*N* = 14 per group). For the Rab35 overexpression experiment, another set of male Wistar rats (17–19 month-old; Charles River Laboratories, Spain) were randomly divided into two groups (*N* = 4–8 per group) and were bilaterally injected in the dorsal hippocampus with the AAV8-GFP or AAV8-Rab-GFP viruses as previously described [10]. Sample sizes for animals were chosen by approximation, assuming that effect sizes would be similar to those in Vaz-Silva et al. [10] and estimating that approximately five animals are required to detect differences between groups. Investigators were not blinded to group allocations during the experiment or when assessing the outcome.

### Western blotting

Western blotting experiments were performed as previously described [10, 21]. Primary antibodies used for western blotting are included in Table 1.

**Table 1:** List of antibodies used in this study.

Antibody	Manufacturer	Concentration
Actin	Abcam (#8224)	1:1000
ACAP2	ProteinTech (#14029-1-AP)	1:1000
APP & CTFs	Biolegend (APP C1/6.1; #802801)	1:1000
BACE1	Cell Signaling (D10E5; #5606)	1:1000
GFP	Invitrogen (#A6455)	1:1000
mCherry	Abcam (#ab125096) Biovision (#5993)	1:1000
OCRL	ProteinTech (#17695-1-AP)	1:1000
Rab5	Synaptic Systems (#108011)	1:1000
Rab7	Abcam (#ab50533)	1:1000
Rab8	ProteinTech (#55296-1-AP)	1:1000
Rab11	Cell Signaling (D4F5; #5589)	1:1000
Rab14	Santa Cruz (#sc-98610)	1:500
Rab35	ProteinTech (#11329-2-AP)	1:1000
Tubulin	Abcam (#ab4074) Sigma (#T9026)	1:5000

### Immunofluorescence microscopy

Immunofluorescence staining in neurons and N2a cells was performed as previously described [21]. Images were acquired using a Zeiss LSM 800 confocal microscope equipped with Airyscan module, using a 63× objective (Plan-Apochromat, NA 1.4; for neurons and N2a cell imaging).

### Neuronal image analyses

Images were analyzed and processed using the Fiji/ImageJ software. PLA puncta were counted using the Multi-point tool, and cell area measured with Polygon selection tool. Colocalization analysis in neurons was performed using the JACoP plugin, in order to obtain the Mander’s coefficient corresponding to the fraction of APP or BACE1 colocalized with each intracellular compartment.

### Retrograde trafficking assay

N2a cells were co-transfected with APP-GFP or FLAG-BACE1 and HA or HA-Rab35 constructs. Approximately 48 h after transfection, cells were starved in serum-free DMEM for 30 min and then subject to antibody feeding using a protocol modified from Vieira et al. and Ubelmann et al. [18, 26]. Briefly, cells were incubated for 30 min at 4 °C with 22C11 or anti-FLAG antibodies diluted 1:100 in complete medium +1 M HEPES, washed with fresh medium, and either immediately fixed with Lorene’s fixative or incubated at 37 °C for 10, 30, or 60 min and then fixed. For immunostaining, cells were permeabilized using Triton X-100 and coverslips immunostained with the following primary + secondary antibody pairs: internalized anti-22C11 or anti-FLAG + goat-anti-mouse Alexa Fluor-568; anti-Syntaxin-6 + Alexa Fluor-647 to tag the trans-Golgi network (TGN); and anti-BACE1 (for BACE1-transfected conditions only) + Alexa Fluor-488 to tag total BACE1. Cells overexpressing Rab35 were detected using anti-HA primary antibody + Alexa Fluor-405 secondary antibody. For analysis, Fiji/ImageJ was used to outline each transfected cell and clear the background. Colocalization analysis between APP or BACE1 and the TGN was determined using the JACoP plugin, with Mander’s coefficient used for reporting the fraction of APP or BACE1 colocalized with the TGN.

### Recycling assay

N2a cells were co-transfected with APP-GFP or FLAG-BACE1 and HA or HA-Rab35 constructs. Forty-eight hours after transfection, cells were starved in serum-free DMEM for 30 min and subjected to an antibody feeding and recycling assay modified from Ubelmann et al. [18]. Briefly, cells were incubated for 30 min at 4 °C with 22C11 antibody or anti-FLAG, coverslips washed with complete medium +1 M HEPES, and cells incubated with goat-anti-mouse unconjugated antibody (1:50; Invitrogen) for 30 min at 4 °C to allow for APP or BACE internalization. Coverslips were then washed with complete medium + HEPES and either fixed with Lorene’s fixative or incubated at 37 °C for 10, 30, or 60 min and then fixed. Coverslips were immunostained with goat-anti-mouse Alexa Fluor-568 for 1 h at RT prior to cell permeabilization to label recycled 22C11 or FLAG antibodies, and any remaining surface antibody was blocked using goat-anti-mouse unconjugated antibody (1:50, 30 min at RT). Following cell permeabilization, internalized 22C11 or FLAG was labeled using goat-anti-mouse Alexa Fluor-647 secondary antibody. Total BACE1 and HA were labeled as in the retrograde trafficking assay. Internalization and recycling of APP and BACE1 were determined using Fiji/ImageJ, by outlining each cell and normalizing the fluorescence of [1] internalized APP or BACE1 and [2] recycled APP or BACE1 to total APP or BACE1. These values were then normalized to the first control timepoint for APP or BACE1 to determine change over time.

### Steady-state surface protein measurement

N2a cells were co-transfected as in the retrograde trafficking and recycling assays. Approximately 48 h after transfection, surface APP and BACE1 were labeled with anti-22C11 or anti-FLAG antibodies prior to cell permeabilization. Following permeabilization, coverslips were immunostained for HA (APP-GFP transfected cells) or HA and BACE1 as described for the retrograde trafficking assay. Fiji/ImageJ was used to determine surface APP and BACE1 by outlining each transfected cell and normalizing the fluorescence of surface APP or BACE1 to total APP or BACE1.

### Bioinformatics analysis

RNA sequencing data across age groups was collected and produced by the Genotype-Tissue Expression (GTEx) project (https://gtexportal.org/home/). Rab35 transcripts per million were normalized to IPO8 transcripts per million for each sample using Microsoft Excel. IPO8 was chosen as a reference gene because it was the most stable gene tested from the GTEx dataset, using RefFinder [27].

### Statistical analysis

Graphing and statistics analysis was performed using Prism (GraphPad). Shapiro–Wilk normality test was used to determine whether data sets were modeled by a normal distribution. Data points detected as outliers were excluded from analyses. Unpaired, two-tailed *t*-tests, one-way ANOVA, or two-way ANOVAs, with appropriate corrections for unequal variances and multiple comparisons, were used with values of *P* < 0.05 considered statistically significant.

## Results

### Rab35 levels are decreased by chronic stress and aging

**Fig. 1:**
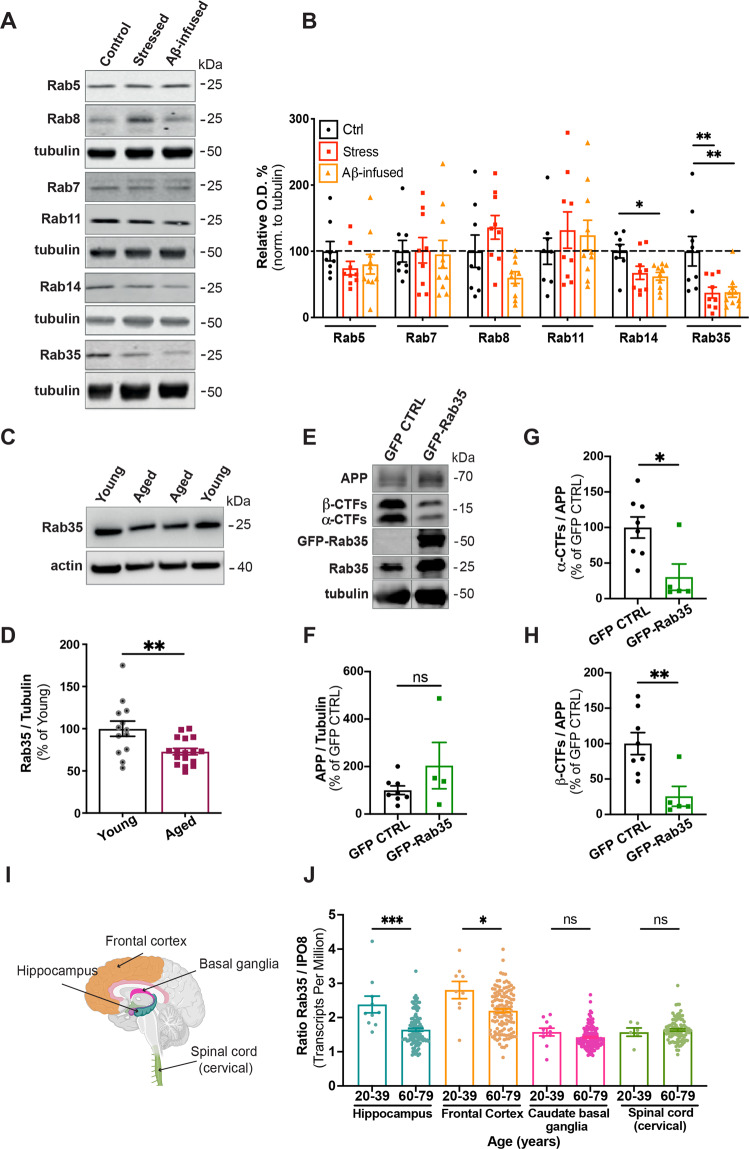
Hippocampal Rab35 levels are decreased by chronic stress and aging. **A**, **B** Representative immunoblots and quantification of Rab protein levels in the hippocampus of control (Ctrl), stressed, and Aβ-infused rats. Blots were probed for the noted Rabs and tubulin, with values normalized to tubulin and expressed as % of the control condition (dotted line)(**P_Rab35 CON vs. stressed_ = 0.0075, *P_Rab14 CON vs. Aβ-infused_ = 0.0228, **P_Rab35 CON vs. Aβ-infused_ = 0.0069; one-way ANOVA, Dunnet post hoc analysis, *n* = 8–10 animals/condition). **C**, **D** Representative immunoblots and quantification of Rab35 levels in the hippocampus of young (4 month old) and aged (22–24 month old) rats. Blots were probed for Rab35 and actin, with values normalized to actin and expressed as % of young animals (**P = 0.0056; unpaired *t*-test, *n* = 13–17/condition). **E**–**H** Representative immunoblots and quantification of full-length APP (**F**) and α-C-terminal and β-C-terminal fragments (CTFs) relative to full-length APP (**G**, **H**) in the dorsal hippocampus of rats bilaterally injected with AAV-GFP or AAV-GFP-Rab35. Blots were probed for APP, Rab35, and tubulin, with values normalized to tubulin and expressed as % of GFP control condition (*P = 0.017, **P = 0.0048; Welch’s unpaired two-tailed *t*-test, *n* = 4–8 animals/condition). **I** Schematic diagram of human brain areas used for the analysis of transcriptome data from the Genotype-Tissue Expression (GTEx) project, created with BioRender. **J** Quantification of Rab35 mRNA transcripts from individuals ages 20–39 years and 60–79 years, normalized to IPO8 (*P = 0.0155, **P = 0.0003; Mann–Whitney test, *n* = 6–135 samples/condition). All numeric data represent mean ± SEM.

We previously showed that high levels of glucocorticoids (GCs) suppress Rab35 expression in hippocampal neurons in vivo [10]. To test whether chronic stress has a similar effect, we measured Rab35 protein levels in hippocampi of 12-month old rats subject to chronic unpredictable stress for 4 weeks. Rab35 levels were decreased by ~60% in stressed rats compared to controls (Fig. 1A, B), and this decrease was not observed for other Rabs associated with endocytic protein trafficking (Fig. 1A, B), in line with our previous findings [10]. In parallel, we monitored hippocampal Rab levels in animals infused with Aβ, a procedure widely used to model early AD neuropathology in rodents and primates [28–30]. Infused Aβ further stimulates Aβ production [5, 31, 32] and circumvents the need to overexpress human APP containing mutation(s) that impact its localization and trafficking. Interestingly, Aβ-infused hippocampi exhibited decreased levels of Rab14 and Rab35, but not other endocytic Rabs (Fig. 1A, B). Since aging is the greatest risk factor for AD, and previous studies report increased amyloidogenic APP processing in the aged brain [33], we also compared Rab35 expression in hippocampi of young (4 month old) versus aged (22–24 month old) rats. Again, we observed a significant (~25%) decrease in hippocampal Rab35 levels in aged animals (Fig. 1C, D), indicating downregulation of Rab35 during aging. To determine whether boosting Rab35 levels could inhibit amyloidogenic processing of APP in aging animals, we injected 17–19 month-old rats with AAV8 to express EGFP or EGFP-Rab35 in the dorsal hippocampus. Intriguingly, Rab35 overexpression decreased the levels of both α and β C-terminal fragments (CTFs) of APP (Fig. 1E, G, H) without altering levels of full-length APP (Fig. 1E, F). These results suggest that Rab35 inhibits APP cleavage, and that its reduction during aging and/or stress may contribute to Aβ overproduction in these conditions.

To investigate whether aging similarly impacts Rab35 levels in human brain, we analyzed gene expression data from the Genotype-Tissue Expression (GTEx) Portal, a collection of data from non-diseased human tissue (https://gtexportal.org/home/). Transcript levels were normalized to those of IPO8, identified by RefFinder [27] as the most stable gene across ages and brain regions of this data set. Intriguingly, Rab35 transcripts were decreased in hippocampus and frontal cortex from individuals 60–79 years of age compared to those 20–39 years of age (Fig. 1I, J). These changes were not seen in basal ganglia or cervical spinal cord, tissues not implicated in stress-related and AD-related pathology (Fig. 1I, J). Together, these findings suggest that Rab35 expression decreases during human aging in the hippocampus and frontal cortex, brain regions impacted by AD and chronic stress [34].

### Rab35 inhibits Aβ production and the endosomal localization of APP and BACE1

**Fig. 2:**
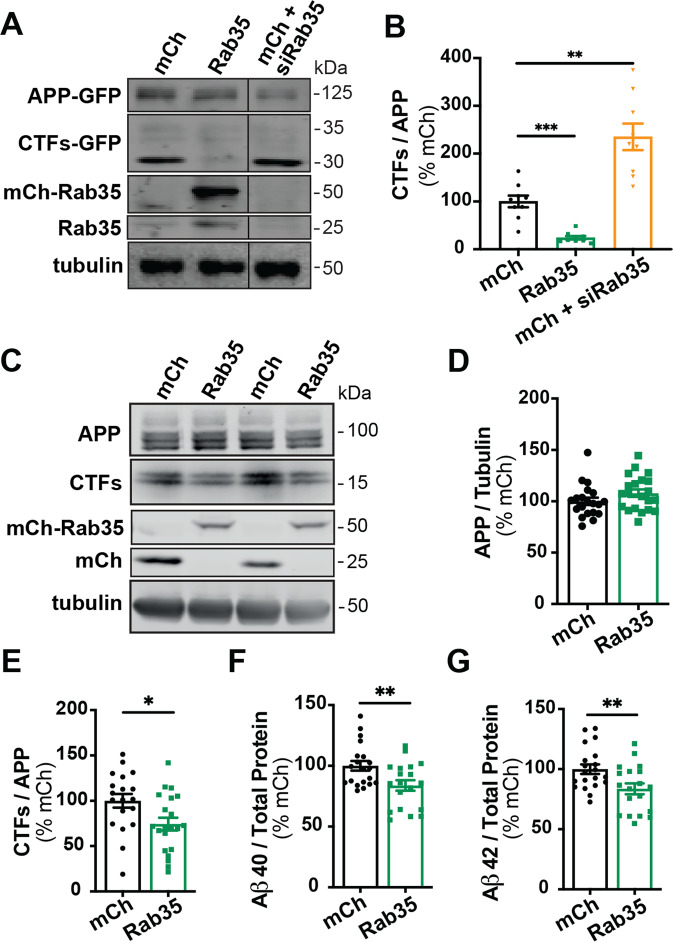
Rab35 expression suppresses APP processing. **A**, **B** Representative immunoblots and quantification of APP CTFs from N2a cells co-expressing APP-GFP with mCherry, mCh-Rab35, or mCh+siRNAs against Rab35 (siRab35). Immunoblots were probed for GFP, tubulin, and Rab35, with values normalized to tubulin and expressed as % of mCh control (***P_mChCtrl vs. mCh-Rab35_ = 0.0006, **P_mChCtrl vs. mCh + siRab35_ = 0.0029; one-way ANOVA with Welch’s correction, *n* = 9 samples/condition, 1 experiment). **C**–**E** Representative immunoblots and quantification of APP CTFs from iPSC-derived cortical neurons expressing mCh or mCh-Rab35. Blots were probed for APP, tubulin, and mCherry, with values normalized to tubulin and expressed as % of mCh control (*P = 0.017; unpaired *t*-test, *n* = 19–21/condition, four independent cultures). **F**, **G** Measurement of Aβ peptides secreted by iPSC-derived human neurons transduced with mCherry or mCh-Rab35. Values are normalized to total protein and expressed as % of mCh control (**P_Aβ40_ = 0.008, **P_Aβ42_ = 0.009; unpaired *t*-test, *n* = 19–20 samples/condition, four independent cultures). All numeric data represent mean ± SEM.

Rab35 was previously identified as a negative regulator of amyloidogenic APP processing in an siRNA screen in non-neuronal cells [19]. To confirm these findings, we measured levels of APP CTFs in N2a cells co-transfected with human APP-GFP and either mCherry, mCh-Rab35, or mCh + siRNAs to knockdown Rab35 (siRab35; see Fig. S1A, B). Overexpression of Rab35 significantly decreased (by ~70%) CTF levels relative to total APP, while Rab35 knockdown significantly increased CTFs (by ~300%; Fig. 2A, B). We confirmed this effect of Rab35 in human induced pluripotent stem cell (iPSC)-derived cortical neurons differentiated by dual inhibition of SMAD signaling [22, 24]. Here, overexpression of mCh-Rab35 did not alter full-length APP levels (Fig. 2C, D) but significantly reduced CTFs (by ~25%; Fig. 2E) as well as Aβ40 and Aβ42 peptides (by 20%; Fig. 2F, G), indicating that Rab35 inhibits APP cleavage and Aβ peptide generation.

**Fig. 3:**
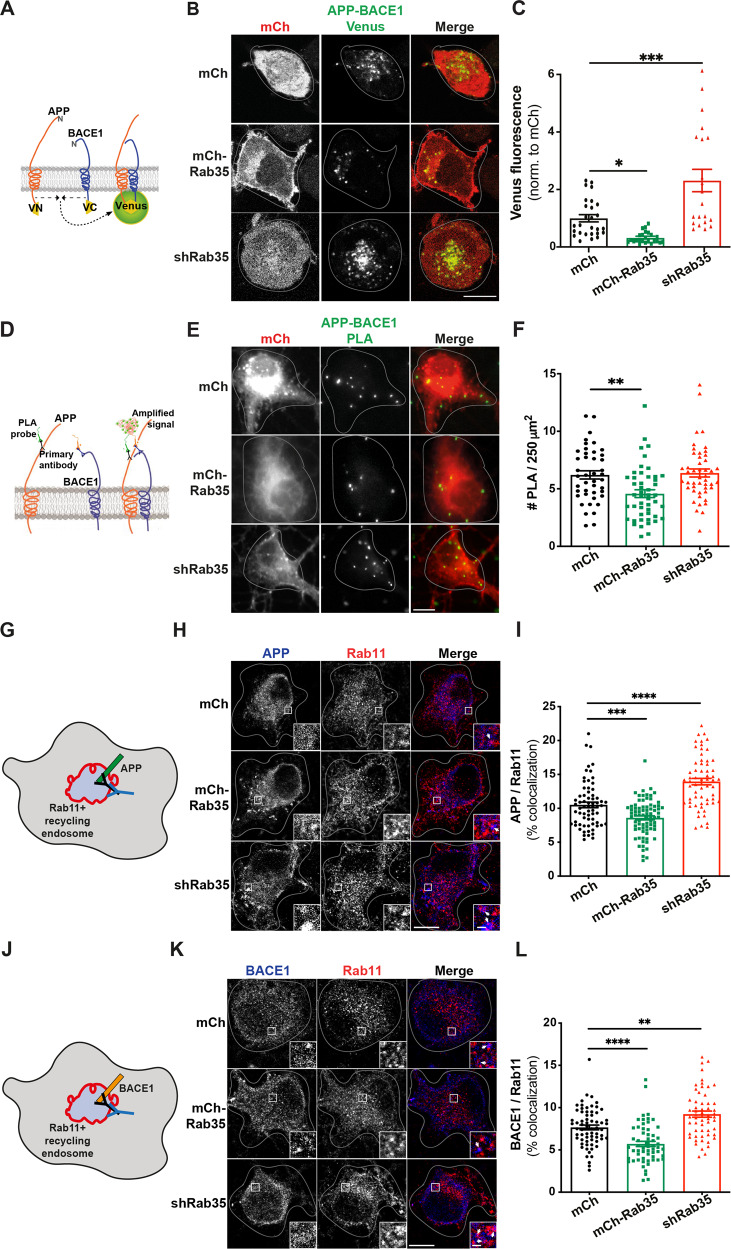
Rab35 negatively regulates APP-BACE1 interaction and endosomal localization in hippocampal neurons. **A** Schematic diagram of bimolecular fluorescence complementation assay, in which APP and BACE1 are tagged with complementary fragments of Venus fluorescent protein (VN and VC, respectively). Reconstitution of Venus fluorescence occurs upon interaction of APP and BACE1. **B**, **C** Representative images and quantification of Venus fluorescence in N2a cells expressing APP:VN, BACE:VC, and either mCh, mCh-Rab35, or shRab35 (*P = 0.042, ***P = 0.0001; one-way ANOVA, Dunnet post-hoc analysis, *n* = 21–27 cells/condition, two experiments). D Schematic diagram of proximity ligation assay (PLA), in which APP and BACE1 are labeled with primary antibodies, which are then recognized by anti-mouse and anti-rabbit PLA probes conjugated to oligonucleotides. When the probes are in close proximity, the oligos become ligated and can be amplified to produce a fluorescent signal marking protein–protein interaction. **E**, **F** Images and quantification for PLA, showing endogenous APP-BACE1 interaction in hippocampal neurons expressing mCh, mCh-Rab35, or shRab35 (**P = 0.001; unpaired student’s *t*-test, *n* = 43–44 cells/condition, two independent cultures). **G** Schematic diagram depicting immunostaining of APP (blue) and Rab11 recycling endosomes (red). **H**, **I** Representative images and quantification of APP colocalization with Rab11 (white arrowheads in insets) in hippocampal neurons expressing mCh, mCh-Rab35, or shRab35 (***P = 0.001, ****P < 0.0001; one-way ANOVA, Dunnet post-hoc analysis, *n* = 62–78 cells/condition, three independent cultures). **J** Schematic diagram depicting immunostaining of BACE1 (blue) and Rab11-positive recycling endosomes (red). **K**, **L** Representative images and quantification of BACE1 colocalization with Rab11 (white arrowheads in insets) in hippocampal neurons expressing mCh, mCh-Rab35, or shRab35 (**P = 0.001, ****P < 0.0001; one-way ANOVA, Dunnett’s post-hoc analysis, *n* = 58–66 cells/condition, three independent cultures). Scale bars: 10 µm; 1 µm for zoomed insets. All numeric data represent mean ± SEM.

To understand how Rab35 regulates APP processing at the subcellular level, we examined its effect on the interaction between APP and BACE1, required for the rate-limiting cleavage of APP into Aβ. As a readout of APP-BACE1 interaction, we utilized a published bimolecular fluorescence complementation (BiFC) assay in which APP and BACE1 are tagged with complementary fragments of Venus fluorescent protein (APP:VN and BACE:VC; Fig. 3A) [35]. Venus fluorescence was measured in N2a cells under control (mCherry), Rab35 overexpression (mCh-Rab35), or Rab35 knockdown (mCh/shRab35 [21]) conditions (Fig. S1C, D). Here, Rab35 overexpression decreased Venus fluorescence by 70% compared to control, suggesting reduced APP–BACE1 interaction, while knockdown increased Venus fluorescence by 200% (Fig. 3B, C). We further assessed the impact of Rab35 overexpression and knockdown on endogenous APP-BACE1 interaction in primary rat hippocampal neurons, using the proximity ligation assay (PLA) in neurons expressing the same mCh-based constructs (Fig. 3D). Consistent with BiFC experiments, Rab35 overexpression significantly decreased PLA puncta density in neuronal cell bodies (from 6 to 4.5 puncta/250 μm^2^; Fig. 3E, F). However, Rab35 knockdown had no effect on PLA puncta density (Fig. 3E, F), suggesting that this assay may not be sensitive enough to detect increases in APP–BACE1 interaction in neurons.

To further evaluate the impact of Rab35 on APP and BACE1 localization in neurons, we examined the effects of Rab35 overexpression/knockdown on their colocalization with Rab11+ recycling endosomes (Fig. 3G, J), a common site of APP cleavage by BACE1 [35, 36]. Rab35 overexpression significantly decreased APP and BACE1 colocalization with Rab11 in cell bodies of hippocampal neurons (by 18% for APP and 25% for BACE1), while Rab35 knockdown increased this colocalization by a similar amount (32% for APP and 21% for BACE1; Fig. 3H, I, K, L). These changes were not due to gross effects of Rab35 overexpression/knockdown on the morphology of Rab11 endosomes (Fig. S1E), and suggest that Rab35 inhibits APP/BACE1 interaction in recycling endosomes by promoting their sorting to other subcellular compartments.

### Rab35 promotes BACE1 trafficking through the retrograde pathway

Given that Rab35 mediates protein degradation through the endolysosomal pathway [10, 21], we first examined whether Rab35 stimulates the degradation of APP and/or BACE1. Using a previously described cycloheximide (CHX)-chase assay [21, 37], we found that Rab35 overexpression or knockdown did not alter the degradation of APP, CTFs, or BACE1 in hippocampal neurons (Fig. S2A–E). Since Rab35 also regulates retrograde trafficking of mannose-6-phosphate receptors from endosomes to the trans-Golgi network (TGN) [11], we next examined whether Rab35 similarly promotes the retrograde trafficking of APP and/or BACE1. Here, we used a modified antibody feeding assay [18, 26] in N2a cells, coupled with immunofluorescence microscopy to monitor the colocalization of internalized APP-GFP or FLAG-BACE1 with the TGN marker syntaxin-6 at several timepoints after antibody labeling (Fig. S3A–D). Rab35 overexpression did not alter APP colocalization with syntaxin-6 at any timepoint compared to the control condition (Fig. S3E, F). To verify that we were primarily tracking full-length APP vs. an N-terminal ectodomain fragment to which our APP antibody (22C11) is raised, we measured colocalization between 22C11 and the C-terminal GFP tag of APP (Fig. S3G). While colocalization of these signals was ~50% (Fig. S3G, H), this measurement significantly underestimated the degree of colocalization due to saturating perinuclear levels of APP-GFP, which obscured dimmer puncta in the periphery where the majority of 22C11 signal was located (Fig. S3G). Moreover, shedding of the APP ectodomain is expected to occur at the plasma membrane [38, 39] and would lead to rapid diffusion of sAPP fragments into the medium rather than their recycling to the TGN. Together, these results indicate that Rab35 does not mediate retrograde trafficking of APP.

**Fig. 4:**
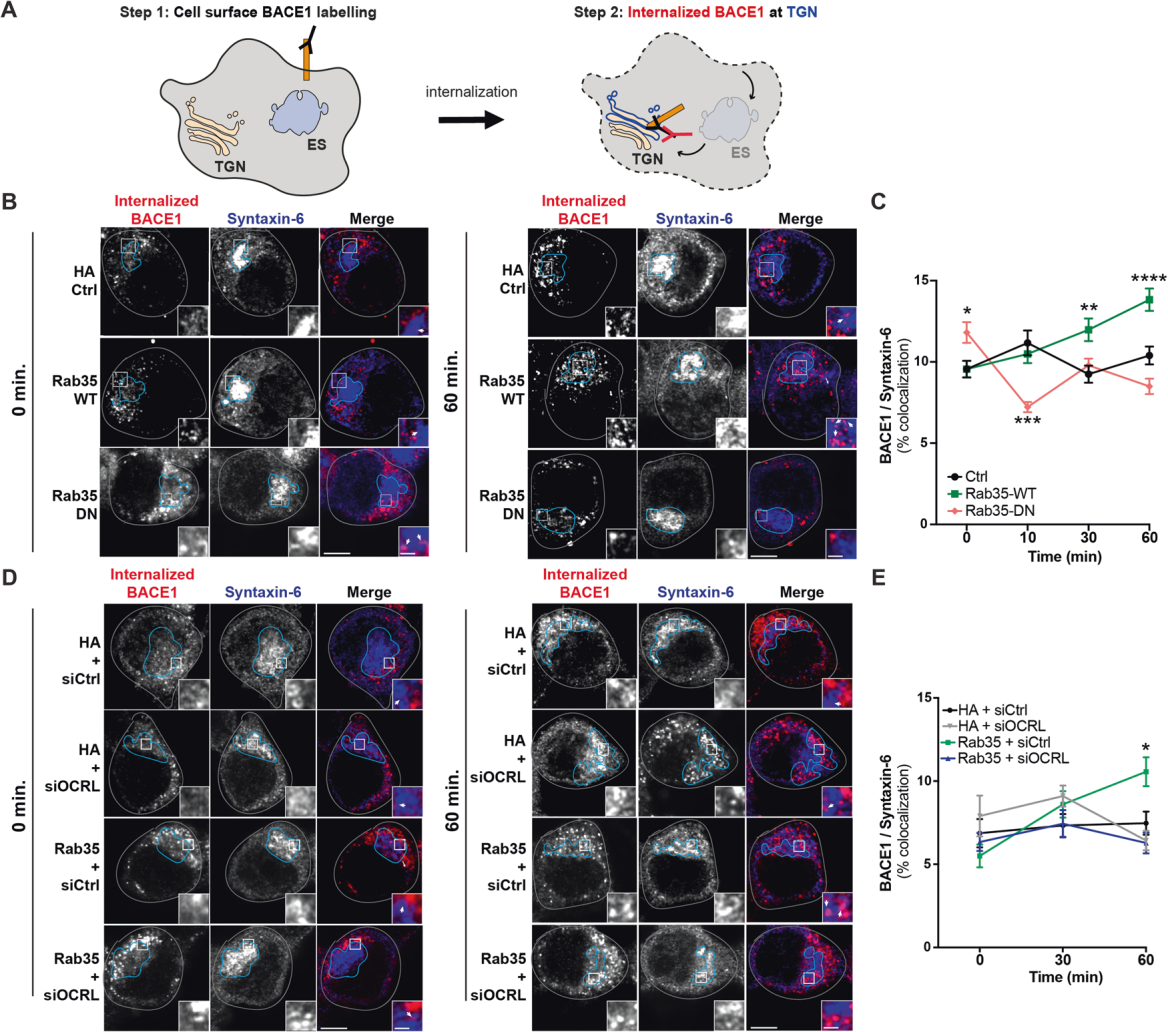
Rab35 stimulates retrograde trafficking of BACE1 via OCRL. **A** Schematic representation of BACE1 internalization assay, in which cell-surface BACE1 was labeled with FLAG antibody, cells were incubated for 0, 10, 30, or 60 min to allow for BACE1 internalization, and finally, cells were immunostained with syntaxin-6 antibodies to label the TGN. **B**, **C** Representative high-resolution images (Zeiss Airyscan) and quantification of BACE1 internalization in N2a cells expressing FLAG-BACE1 and either HA Control, HA-Rab35 wild-type (WT), or HA-Rab35 dominant-negative (DN). Internalized BACE1 (red) and syntaxin-6 (blue) are shown at 0 and 60 min time points post-labeling, with cells outlined in gray, the TGN outlined in blue, and white arrowheads pointing to areas of colocalization in insets (*P_DN-0 min_ = 0.0392, **P_DN-10 min_ = 0.0011, **P_WT-30 min_ = 0.0025, ****P_WT-60 min_ < 0.0001; 2-way ANOVA and Sidak post hoc analysis, *n* = 45–155 cells per condition/timepoint, 2–5 experiments. Time × Rab35 interaction *F*_6,1253_ = 8.002, *P* < 0.0001, overall *Rab35* effect *F*_2,1253_ = 10.43, *P* < 0.0001). **D**, **E** Representative high-resolution images (Zeiss Airyscan) and quantification of BACE1 internalization in N2a cells expressing FLAG-BACE1 and HA or HA-Rab35 together with control siRNA (siCtrl) or siRNA to knockdown OCRL (siOCRL). Internalized BACE1 (red) and syntaxin-6 (blue) are shown at 0 and 60 min time points post-labeling (*P_HA+siCtrl vs. Rab35+siCtrl_ = 0.0151, 2-way ANOVA and Tukey’s multiple comparisons test, *n* = 50–61 cells per condition/time point, two experiments. Time × Condition interaction *F*_6,635_ = 3.861, *P* = 0.009). Scale bars: 5 µm; 1 μm for zoomed insets. All numeric data represent mean ± SEM.

We next performed the same assay with FLAG-BACE1 (Fig. 4A). In contrast to the lack of effect on APP, we found that Rab35 overexpression significantly increased the colocalization of internalized BACE1 with syntaxin-6 at the 30 and 60 min timepoints post-labeling (Fig. 4B–E), indicating an effect on retrograde trafficking of BACE1. To determine whether this effect requires Rab35 activation, we performed the same assay in the presence of dominant-negative (DN) HA-Rab35 to mimic loss-of-function. Although DN Rab35 expression was lower than that of WT Rab35 (Fig. S4A, B), suggesting that our results likely underestimated its effects on BACE1 retrograde trafficking, we found that DN Rab35 either reduced or had no impact on BACE1/syntaxin-6 colocalization compared to the control condition (Fig. 4B, C). These findings indicate that Rab35 activation is required for stimulating BACE1 retrograde trafficking.

The Rab35 effector and lipid phosphatase OCRL (Oculocerebrorenal Syndrome of Lowe Inositol Polyphosphate-5-Phosphatase) is required downstream of Rab35 for the retrograde trafficking of mannose-6-phosphate receptors [11]. To test whether Rab35-mediated retrograde trafficking of BACE1 also requires OCRL, we performed the antibody feeding/TGN colocalization assay in the presence of siRNAs against OCRL (siOCRL; see Fig. S4C, D). While OCRL knockdown alone did not affect BACE1 colocalization with syntaxin-6 compared to control siRNAs, it did prevent the Rab35-mediated increase in BACE1 retrograde trafficking (Fig. 4D, E), indicating its necessity for this trafficking pathway.

To investigate whether Rab35 similarly alters BACE1 trafficking in hippocampal neurons, we measured endogenous APP and BACE1 colocalization with syntaxin-6 following Rab35 overexpression and knockdown. Consistent with our data in N2a cells, APP/syntaxin-6 colocalization was not affected by either manipulation (Fig. S4E, F), while BACE1/syntaxin-6 colocalization was significantly increased by Rab35 overexpression (Fig. S4G, H). This increase was not caused by Rab35-mediated alterations in TGN morphology, as the density and size of syntaxin-6 puncta were unchanged by Rab35 overexpression and knockdown (Fig. S4I, J).

### Rab35 stimulates APP recycling to the plasma membrane

**Fig. 5:**
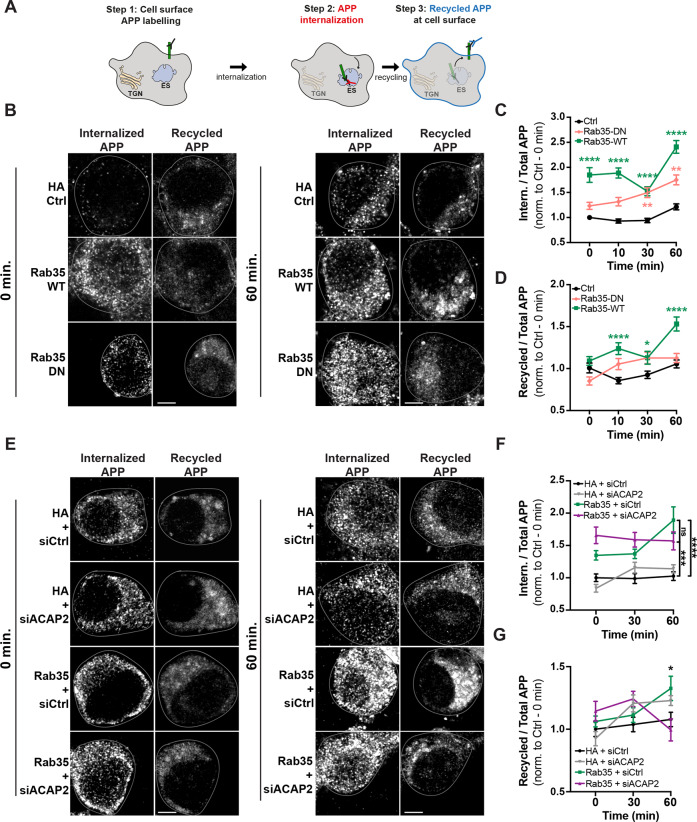
Rab35 stimulates APP recycling to the plasma membrane via ACAP2. **A** Schematic representation of the APP recycling assay, in which APP internalization and recycling were assessed by labeling cell-surface APP with 22C11 antibody followed by cell incubation for 0, 10, 30, or 60 min, and fixation and immunostaining with secondary antibodies to detect recycled or internalized APP. **B**–**D** Representative images and quantification of APP internalization and recycling in N2a cells expressing APP-GFP and either HA control, HA-Rab35 WT, or HA-Rab35 DN. Internalized and recycled APP are shown at 0 and 60 min time points post-labeling, with cells outlined in gray (*P_WT-30 min_ = 0.0299, **P_DN-30 min_ = 0.0027, **P_DN-60 min_ = 0.0017, ****P < 0.0001, 2-way ANOVA and Sidak post-hoc analysis, *n* = 53–269 cells per condition/timepoint, 2–5 experiments. For APP internalization: Time × Rab35 interaction *F*_6,1897_ = 3.002, *P* = 0.0064, overall *Rab35* effect *F*_2,1897_ = 118.9. For APP recycling: Time × Rab35 interaction *F*_6,1897_ = 3.443, *P* = 0.0022, overall *Rab35* effect *F*_2,1897_ = 26.03). **E**–**G** Representative images and quantification of APP internalization and recycling in N2a cells expressing APP-GFP and either HA or HA-Rab35, together with control siRNA (siCtrl) or siRNA to knockdown ACAP2 (siACAP2). Internalized and recycled APP are shown at 0 and 60 min time points post-labeling (for **F** ***P_HA+siCtrl vs. Rab35+siACAP2_ = 0.0003, ****P_HA+siCtrl vs. Rab35+siCtrl_ < 0.0001, 2-way ANOVA and Tukey’s multiple comparisons test, *n* = 20–48 cells per condition/timepoint, three experiments. *Time* × *Rab35* interaction *F*_4,310_ = 2.843, *P* = 0.0244, overall *Rab35/ACAP2* effect *F*_2,310_ = 39.12, *P* < 0.0001. **G** *P_HA+siCtrl vs. Rab35+siCtrl_ = 0.0457, *P*_HA+siCtrl vs. Rab35+siACAP2_ = 0.66, two-way ANOVA with Tukey’s multiple comparisons test, *n* = 20–48 cells per condition/timepoint, three experiments. *Time* × *Condition* interaction *F*_4,310_ = 2.877, *P* = 0.023, overall *Condition* effect *F*_2,310_ = 3.279, *P* = 0.039). Scale bars: 5 µm. All numeric data represent mean ± SEM.

Rab35’s ability to promote BACE1 trafficking to the TGN could be sufficient for reducing APP amyloidogenic cleavage. However, Rab35 also facilitates the fast recycling of cell-surface proteins (e.g., T-cell receptors, β1 integrin) between endosomes and the plasma membrane (PM) in a pathway that operates in parallel with Rab11+ recycling [12–14]. Stimulating APP and/or BACE1 trafficking into this pathway would also reduce their localization in Rab11+ endosomes. To determine whether Rab35 promotes the fast recycling of APP and/or BACE1, we used another antibody feeding assay [18] to monitor the internalization and PM recycling of APP-GFP and FLAG-BACE1 at four timepoints (Fig. S5A–D and Fig. 5A). Intriguingly, Rab35 overexpression stimulated both APP internalization and recycling to the PM at nearly all post-labeling timepoints (10, 30, and 60 min) compared to the control condition (Fig. 5B–D). Rab35 also stimulated BACE1 internalization at earlier timepoints (0 and 10 min; Fig. S5E, F) but did not alter BACE1 recycling to the PM (Fig. S5E, G). Consistent with these findings, we observed a 40% increase in cell-surface levels of APP, but not BACE1, in N2a cells expressing Rab35 (Fig. S5H–K).

To determine whether APP internalization and recycling were dependent on Rab35 activation, we performed this assay in the presence of DN Rab35. As anticipated, expression of DN Rab35 did not stimulate APP recycling relative to the control condition (Fig. 5B, D), indicating the dependence of this trafficking step on Rab35 activation. Surprisingly, DN Rab35 did stimulate APP internalization at the 30 and 60 min timepoints to a similar degree as wild-type Rab35 (Fig. 5B, C), suggesting that Rab35 mediates APP internalization independently of its activation state.

Fast endocytic recycling has been shown to occur through two distinct pathways, mediated by Rab35 effectors OCRL and ACAP2 [40]. Using our internalization/recycling assay in combination with OCRL siRNAs, we found that OCRL knockdown did not alter Rab35-induced APP internalization (Fig. S6A, B), but further stimulated Rab35-induced APP recycling at the 60 min timepoint (by ~50%; Fig. S6A, C). These findings suggest that OCRL knockdown frees Rab35 to interact with a different effector responsible for APP recycling, thereby enhancing this trafficking event. We next tested whether this effector is ACAP2, again using the antibody feeding assay in combination with ACAP2 siRNAs (Fig. S6D, E). ACAP2 knockdown did not alter APP internalization compared to siRNA control, nor mitigate Rab35-mediated APP internalization (Fig. 5E, F), as expected if this sorting step is independent of Rab35 activation. However, ACAP2 knockdown abolished Rab35-induced APP recycling to the PM at the 60 min timepoint (Fig. 5E, G). These findings demonstrate that Rab35 stimulates APP sorting into the fast recycling pathway, via GTP-independent stimulation of APP internalization and ACAP2-dependent stimulation of APP recycling to the PM. Finally, we tested whether ACAP2, like OCRL, has a role in Rab35-mediated BACE1 retrograde trafficking. We found that knockdown of ACAP2 did not alter the Rab35-mediated increase in BACE1 colocalization with syntaxin-6 (Fig. S6F, G), confirming that Rab35 regulates APP and BACE1 trafficking via distinct mechanisms.

### Rab35 counteracts GC-induced amyloidogenic trafficking of APP and BACE1

**Fig. 6:**
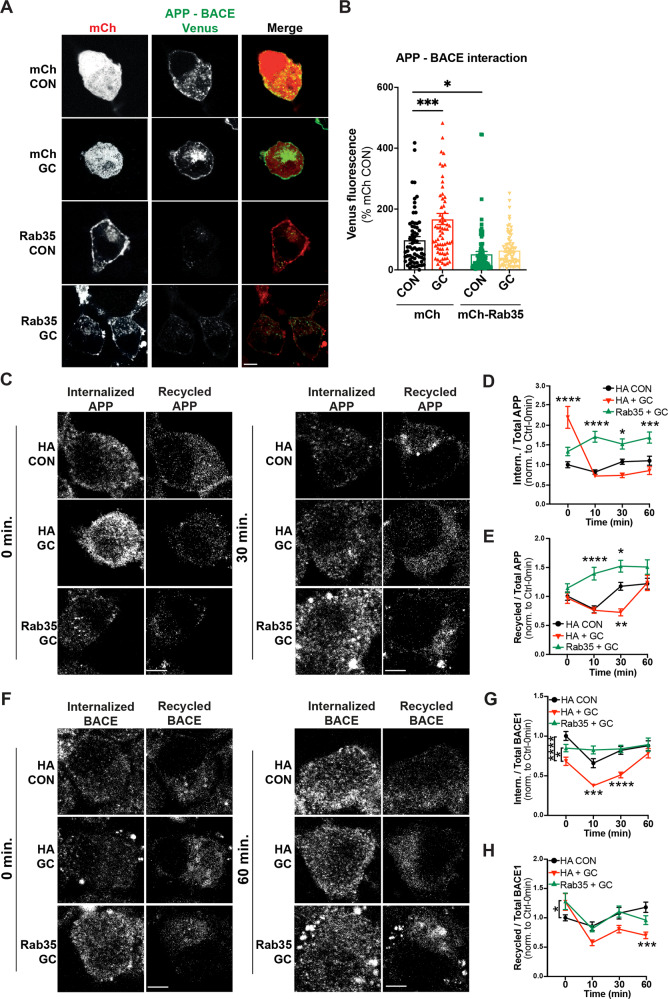
GC-induced amyloidogenic APP/BACE1 trafficking is blocked by Rab35 overexpression. **A**, **B** Representative images and quantification of Venus fluorescence intensity in N2a cells expressing APP:VN, BACE:VC, and either mCh or mCh-Rab35, treated with GC or vehicle control (***P_mCh CON vs. mCh GC_ = 0.0004, *P_mCh CON vs. mCh-Rab35 CON_ = 0.028, two-way ANOVA, Tukey post-hoc analysis, *n* = 3 experiments. Rab35 × GC interaction *F*_1,291_ = 5.989, *P* = 0.015, overall *Rab35* effect *F*_1,291_ = 41.35, *P* < 0.0001, overall *GC* effect *F*_1,291_ = 12.19, *P* = 0.0006). Scale bar: 10 µm. **C**–**E** Representative images and quantification of APP internalization and recycling in N2a cells expressing APP-GFP and either HA or HA-Rab35, treated with GC or vehicle control (for **D** *P_HA+DMSO vs. HA-Rab35+GC_ = 0.0119, ***P_HA+DMSO vs. HA-Rab35+GC_ = 0.0009, ****P < 0.0001; two-way ANOVA with Tukey’s post hoc analysis, *n* = 47–77 cells per condition/timepoint, three experiments. For APP internalization: Time × Condition interaction *F*_6,780_ = 15.81, *P* < 0.0001. **E** ****P_HA+DMSO vs. HA-Rab35+GC_ < 0.0001, *P_HA+DMSO vs. HA-Rab35+GC_ = 0.0136, **P_HA+DMSO vs. HA+GC_ = 0.0011; two-way ANOVA with Tukey’s post hoc analysis, *n* = 47–77 cells per condition/timepoint. For APP recycling: Time × Condition interaction *F*_6,759_ = 3.556, *P* = 0.0013). **F**–**H** Representative images and quantification of BACE1 internalization and recycling in N2a cells expressing FLAG-BACE1 and either HA or HA-Rab35, treated with GCs or vehicle control (for **G** *P_HA+GC vs. HA-Rab35+GC_ = 0.0491, ***P_HA+DMSO vs. HA-+GC_ = 0.0003, ****P < 0.0001; two-way ANOVA with Tukey’s multiple comparisons test, *n* = 54–76 cells per condition/timepoint, three experiments. For BACE1 internalization: Time × Condition interaction *F*_6,765_ = 3.385, *P* = 0.0027. **H** *P_HA+DMSO vs. HA-Rab35+GC_ = 0.0487, ***P_HA+DMSO vs. HA+GC_ = 0.0009; two-way ANOVA with Tukey’s multiple comparisons test, *n* = 54–76 cells per condition/timepoint, three experiments. For BACE1 recycling: Time × Condition interaction *F*_6,762_ = 3.504, *P* = 0.0020). Scale bars: 5 µm. All numeric data represent mean ± SEM.

Although chronic stress and high glucocorticoid (GC) levels are known to stimulate Aβ production in vitro and in vivo [5, 41, 42], their effects on APP and BACE1 intracellular trafficking are largely unexplored. We therefore examined whether high GC levels alter the interaction between APP and BACE1 within endosomes, again using the Venus BiFC assay in N2a cells. Treatment with the synthetic GC dexamethasone (10 µM) significantly increased Venus intensity, indicating that exposure to high GCs promotes APP-BACE1 interaction (Fig. 6A, B). We next examined whether overexpression of Rab35 could block this effect, as predicted if GC-induced downregulation of Rab35 underlies the increased APP-BACE1 interaction. Indeed, Rab35 overexpression blocked the GC-induced increase in Venus intensity (Fig. 6A, B), suggesting that Rab35 prevents this pro-amyloidogenic interaction between APP and BACE1.

We next tested whether exposure to high GC levels impacts the APP and BACE1 trafficking pathways mediated by Rab35. Using the aforementioned antibody feeding assays, we found that 24 h GC treatment did not alter BACE1 retrograde trafficking (Fig. S7A, B), but significantly altered the kinetics of APP and BACE1 internalization and recycling from the PM. In particular, GCs stimulated APP internalization at the 0 min timepoint and decreased its recycling back to the PM at the 30 min timepoint (Fig. 6C–E), while inhibiting BACE1 internalization and recycling to the PM at several timepoints (Fig. 6F–H). Remarkably, Rab35 overexpression prevented these GC-induced trafficking deficits and even enhanced APP recycling compared to the control condition (Fig. 6C, E), similar to its actions in the absence of GCs (see Fig. 5). Together, these findings demonstrate that GCs disrupt the endocytic trafficking of APP and BACE1, resulting in their increased colocalization, and that Rab35 overexpression rescues these GC-driven effects.

## Discussion

**Fig. 7:**
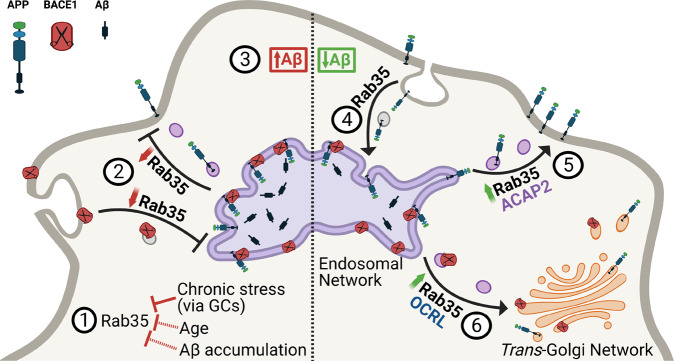
Working model summarizing the interplay between Rab35 and stress/GC on APP trafficking and processing. AD etiopathological factors such as advanced age, Aβ accumulation, or exposure to chronic stress and/or high levels of glucocorticoids (GC), lead to a significant reduction of Rab35 levels [1]. This reduction inhibits APP and BACE1 recycling with the plasma membrane [2], increasing APP-BACE1 association in the endosomal network and stimulating amyloidogenic APP processing and Aβ production [3]. Alternatively, high levels of Rab35 promote APP internalization [4] and recycling to the plasma membrane via the effector ACAP2 [5], and in parallel stimulate BACE1 retrograde trafficking to the trans-Golgi network via the effector OCRL [6]. These trafficking events reduce APP-BACE1 interaction within the endosomal network, thereby decreasing production of APP CTFs and Aβ.

It has been unclear how chronic stress and high GC levels alter cellular trafficking pathways to precipitate amyloidogenesis and Tau pathology. Our studies indicate that GC-induced downregulation of Rab35, a master regulator of endosomal trafficking, may contribute to both events. We previously found that Rab35 mediates Tau degradation via the endolysosomal pathway, and that its GC-driven transcriptional suppression leads to Tau accumulation in the hippocampus, inducing synaptic loss and dendritic atrophy [10]. In the current study, we show for the first time that Rab35 regulates APP and BACE1 trafficking (Fig. 7), and that its suppression by GCs leads to increased interaction between APP and BACE1 within the endosomal network, likely facilitating Aβ overproduction. Importantly, Rab35 overexpression protects against this GC-driven interaction (see Fig. 7), supporting the concept that Rab35 downregulation is a precipitating factor in stress/GC-induced amyloid pathology.

APP misprocessing and overproduction of Aβ are regarded as triggering events for AD pathogenesis, underscoring the need to elucidate the mechanisms of amyloidogenic APP processing. Importantly, previous studies show that Aβ is not the only toxic component of the amyloidogenic pathway, as APP β-CTFs (the intracellular product of BACE1 cleavage) have intrinsic neurotoxic properties and can promote Tau hyperphosphorylation and accumulation independently of Aβ, leading to synaptic degeneration and impaired cognition [43]. Targeting the molecular modulators of APP and/or BACE1 intracellular trafficking could thus serve as a promising therapeutic approach against amyloidogenesis [44]. Defects in the endolysosomal pathway are the earliest cellular feature of AD [17, 45], suggesting that dysfunction of endosomal trafficking underlies AD pathogenesis. Indeed, endosomes are the major sites of Aβ production, and conditions associated with decreased residence of APP and/or BACE1 in these compartments typically inhibit Aβ generation [17]. Here, we demonstrate that Rab35 decreases the localization of both APP and BACE1 in recycling endosomes (Fig. 7). For APP, this decrease occurs via stimulation of its recycling to the PM through ACAP2, similar to Rab35 stimulation of the endocytic recycling of other PM-associated proteins [12–14]. Not only does this fast recycling pathway decrease APP colocalization with BACE1 in endosomes, but it also boosts levels of APP at the PM (Fig. S4), likely promoting cleavage by α-secretase and thus preventing amyloidogenic processing. For BACE1, the Rab35-mediated decrease in endosomal sorting results from its retrograde trafficking to the TGN through OCRL [11]. Interestingly, two genes identified as risk factors for late-onset AD (LOAD), namely VPS35 and VPS26, encode proteins that regulate the retrograde pathway [46]. Mutation of these genes is hypothesized to disrupt APP retrograde trafficking to the TGN, increasing the APP-BACE1 interaction in endosomes and therefore stimulating Aβ production [47]. Although we did not see any effect of Rab35 on the retrograde trafficking of APP, Rab35 stimulation of BACE1 retrograde trafficking should similarly reduce APP–BACE1 interaction within endosomes. Rab35 could also regulate BACE1 trafficking through Arf6, another small GTPase previously reported to mediate BACE1 endocytosis from the PM into the endosomal network [48]. Rab35 and Arf6 are known to inhibit one another’s activation in a variety of cellular processes [13, 49]; thus, Rab35 overexpression could reduce BACE1 endocytosis via Arf6 inhibition. However, we find that cell-surface BACE1 levels are unchanged by Rab35 overexpression, suggesting that Rab35 does not inhibit BACE1 endocytosis. Future studies should clarify the Arf6-Rab35 signaling relationship in the context of APP and BACE1 trafficking.

Another intriguing finding of our study is that Rab35 levels are decreased by several AD etiopathological factors including advanced age, Aβ accumulation, and exposure to chronic stress and/or high levels of glucocorticoids. Aging is the greatest risk factor for AD, and studies have linked aging to increased amyloidogenic APP processing [33], although the underlying mechanism(s) of APP misprocessing in aged brain remain unclear. We demonstrate here that Rab35 protein levels are reduced in hippocampus of aged vs. young rats, and that Rab35 overexpression protects against amyloidogenic APP cleavage in aging animals. These findings are in line with human transcriptomic data in which Rab35 transcripts are reduced in hippocampus and cortex of older (60–79 year old) individuals compared to younger (20–39 year old) ones. Importantly, reduced Rab35 levels are also observed in hippocampus following Aβ infusion, an experimental model of AD that mimics early AD neuropathology and further stimulates amyloidogenic APP processing [5, 31, 32]. In addition to our own observations, other studies have reported that Rab35 levels are reduced under conditions associated with AD risk. For instance, decreased Rab35 levels were observed in the brains of mice expressing the human ApoE4 allele [50], the strongest genetic risk factor for LOAD. ApoE4 carriers have a significantly increased probability of developing AD and exhibit greater accumulation of Aβ in their brains compared to carriers of other ApoE alleles [51]. Another recent human study reported decreased Rab35 levels in brain-derived exosomes from athletes following mild traumatic brain injury (TBI) [52], known to elicit AD-like neuropathology and predispose to AD. Together, these studies support a role for Rab35 in APP misprocessing precipitated by stress, aging, and AD-related conditions.

Finally, our current in vitro and in vivo studies show that high GC levels and/or chronic stress reduce Rab35 levels and stimulate the pro-amyloidogenic trafficking of APP and BACE1, while Rab35 overexpression attenuates these effects. These findings are in line with previous work demonstrating that chronic stress/GCs trigger APP misprocessing and Aβ overproduction [5, 41, 42], and offer a novel, Rab35-linked mechanism to explain how stress/GCs precipitate amyloidogenesis. Combined with our earlier study showing the critical role of Rab35 in Tau degradation [10], these findings suggest that Rab35 may be the molecular link through which chronic stress via GCs trigger both major AD pathomechanisms: Aβ overproduction and Tau accumulation [4, 5, 9, 41]. Altogether, our findings support multiple roles for Rab35 in the trafficking of AD-relevant proteins, and suggest that its downregulation may precipitate amyloidogenesis. These studies highlight Rab35’s potential relevance for AD brain pathology and suggest that additional work investigating its roles in AD-related intracellular pathways could open novel therapeutic avenues for treating AD.

## Supplementary information

Supplemental Figure Legends

Supplemental Figures

## Data Availability

The data generated and analyzed in this study are available from the corresponding author upon reasonable request.

## References

[1] Querfurth HW, LaFerla FM (2010). Alzheimer’s disease. N Engl J Med.

[2] Machado A, Herrera AJ, de Pablos RM, Espinosa-Oliva AM, Sarmiento M, Ayala A (2014). Chronic stress as a risk factor for Alzheimer’s disease. Rev Neurosci.

[3] Mravec B, Horvathova L, Padova A (2018). Brain under stress and Alzheimer’s disease. Cell Mol Neurobiol.

[4] Vyas S, Rodrigues AJ, Silva JM, Tronche F, Almeida OF, Sousa N (2016). Chronic stress and glucocorticoids: from neuronal plasticity to neurodegeneration. Neural Plast.

[5] Catania C, Sotiropoulos I, Silva R, Onofri C, Breen KC, Sousa N (2009). The amyloidogenic potential and behavioral correlates of stress. Mol Psychiatry.

[6] Sotiropoulos I, Catania C, Pinto LG, Silva R, Pollerberg GE, Takashima A (2011). Stress acts cumulatively to precipitate Alzheimer’s disease-like tau pathology and cognitive deficits. J Neurosci.

[7] Kulstad JJ, McMillan PJ, Leverenz JB, Cook DG, Green PS, Peskind ER (2005). Effects of chronic glucocorticoid administration on insulin-degrading enzyme and amyloid-beta peptide in the aged macaque. J Neuropathol Exp Neurol.

[8] Pinheiro S, Silva J, Mota C, Vaz-Silva J, Veloso A, Pinto V (2015). Tau mislocation in glucocorticoid-triggered hippocampal pathology. Mol Neurobiol..

[9] Lopes S, Vaz-Silva J, Pinto V, Dalla C, Kokras N, Bedenk B (2016). Tau protein is essential for stress-induced brain pathology. Proc Natl Acad Sci USA.

[10] Vaz-Silva J, Gomes P, Jin Q, Zhu M, Zhuravleva V, Quintremil S (2018). Endolysosomal degradation of Tau and its role in glucocorticoid-driven hippocampal malfunction. EMBO J.

[11] Cauvin C, Rosendale M, Gupta-Rossi N, Rocancourt M, Larraufie P, Salomon R (2016). Rab35 GTPase triggers switch-like recruitment of the Lowe syndrome lipid phosphatase OCRL on newborn endosomes. Curr Biol.

[12] Argenzio E, Margadant C, Leyton-Puig D, Janssen H, Jalink K, Sonnenberg A (2014). CLIC4 regulates cell adhesion and beta1 integrin trafficking. J Cell Sci.

[13] Allaire PD, Seyed Sadr M, Chaineau M, Seyed Sadr E, Konefal S, Fotouhi M (2013). Interplay between Rab35 and Arf6 controls cargo recycling to coordinate cell adhesion and migration. J Cell Sci.

[14] Patino-Lopez G, Dong X, Ben-Aissa K, Bernot KM, Itoh T, Fukuda M (2008). Rab35 and its GAP EPI64C in T cells regulate receptor recycling and immunological synapse formation. J Biol Chem.

[15] Haass C, Kaether C, Thinakaran G, Sisodia S (2012). Trafficking and proteolytic processing of APP. Cold Spring Harb Perspect Med.

[16] Bera S, Camblor-Perujo S, Calleja Barca E, Negrete-Hurtado A, Racho J, De Bruyckere E (2020). AP-2 reduces amyloidogenesis by promoting BACE1 trafficking and degradation in neurons. EMBO Rep.

[17] Small SAS-S S, Mayeux R, Petsko GA (2017). Endosomal traffick jams represent a pathogenic hub and therapeutic target in Alzheimer’s disease. Trends Neurosci.

[18] Ubelmann F, Burrinha T, Salavessa L, Gomes R, Ferreira C, Moreno N (2017). Bin1 and CD2AP polarise the endocytic generation of beta-amyloid. EMBO Rep.

[19] Udayar V, Buggia-Prevot V, Guerreiro RL, Siegel G, Rambabu N, Soohoo AL (2013). A paired RNAi and RabGAP overexpression screen identifies Rab11 as a regulator of beta-amyloid production. Cell Rep.

[20] Kiral FR, Kohrs FE, Jin EJ, Hiesinger PR (2018). Rab GTPases and membrane trafficking in neurodegeneration. Curr Biol.

[21] Sheehan P, Zhu M, Beskow A, Vollmer C, Waites CL (2016). Activity-dependent degradation of synaptic vesicle proteins requires Rab35 and the ESCRT pathway. J Neurosci.

[22] Chambers SM, Fasano CA, Papapetrou EP, Tomishima M, Sadelain M, Studer L (2009). Highly efficient neural conversion of human ES and iPS cells by dual inhibition of SMAD signaling. Nat Biotechnol.

[23] Topol A, Tran NN, Brennand KJ (2015). A guide to generating and using hiPSC derived NPCs for the study of neurological diseases. J Vis Exp.

[24] Pre D, Nestor MW, Sproul AA, Jacob S, Koppensteiner P, Chinchalongporn V (2014). A time course analysis of the electrophysiological properties of neurons differentiated from human induced pluripotent stem cells (iPSCs). PLoS ONE.

[25] Paxinos G, Watson C, Pennisi M, Topple A (1985). Bregma, lambda and the interaural midpoint in stereotaxic surgery with rats of different sex, strain and weight. J Neurosci Methods.

[26] Vieira SI, Rebelo S, Esselmann H, Wiltfang J, Lah J, Lane R (2010). Retrieval of the Alzheimer’s amyloid precursor protein from the endosome to the TGN is S655 phosphorylation state-dependent and retromer-mediated. Mol Neurodegener.

[27] Xie F, Xiao P, Chen D, Xu L, Zhang B (2012). miRDeepFinder: a miRNA analysis tool for deep sequencing of plant small RNAs. Plant Mol Biol.

[28] Frautschy SA, Yang F, Calderon L, Cole GM (1996). Rodent models of Alzheimer’s disease: rat A beta infusion approaches to amyloid deposits. Neurobiol Aging.

[29] Geula C, Wu CK, Saroff D, Lorenzo A, Yuan M, Yankner BA (1998). Aging renders the brain vulnerable to amyloid beta-protein neurotoxicity. Nat Med.

[30] Stephan A, Phillips AG (2005). A case for a non-transgenic animal model of Alzheimer’s disease. Genes Brain Behav.

[31] Heredia L, Lin R, Vigo FS, Kedikian G, Busciglio J, Lorenzo A (2004). Deposition of amyloid fibrils promotes cell-surface accumulation of amyloid beta precursor protein. Neurobiol Dis.

[32] Lorenzo A, Yuan M, Zhang Z, Paganetti PA, Sturchler-Pierrat C, Staufenbiel M (2000). Amyloid beta interacts with the amyloid precursor protein: a potential toxic mechanism in Alzheimer’s disease. Nat Neurosci.

[33] Kimura N, Samura E, Suzuki K, Okabayashi S, Shimozawa N, Yasutomi Y (2016). Dynein dysfunction reproduces age-dependent retromer deficiency: concomitant disruption of retrograde trafficking is required for alteration in beta-amyloid precursor protein metabolism. Am J Pathol.

[34] Sotiropoulos I, Silva JM, Gomes P, Sousa N, Almeida OFX (2019). Stress and the etiopathogenesis of Alzheimer’s disease and depression. Adv Exp Med Biol.

[35] Das U, Wang L, Ganguly A, Saikia JM, Wagner SL, Koo EH (2016). Visualizing APP and BACE-1 approximation in neurons yields insight into the amyloidogenic pathway. Nat Neurosci.

[36] Tan JZA, Gleeson PA (2019). The role of membrane trafficking in the processing of amyloid precursor protein and production of amyloid peptides in Alzheimer’s disease. Biochim Biophys Acta.

[37] Miranda AM, Lasiecka ZM, Xu Y, Neufeld J, Shahriar S, Simoes S (2018). Neuronal lysosomal dysfunction releases exosomes harboring APP C-terminal fragments and unique lipid signatures. Nat Commun.

[38] Carey RM, Balcz BA, Lopez-Coviella I, Slack BE (2005). Inhibition of dynamin-dependent endocytosis increases shedding of the amyloid precursor protein ectodomain and reduces generation of amyloid beta protein. BMC Cell Biol.

[39] Lichtenthaler SF (2006). Ectodomain shedding of the amyloid precursor protein: cellular control mechanisms and novel modifiers. Neurodegener Dis.

[40] Mrozowska PS, Fukuda M (2016). Regulation of podocalyxin trafficking by Rab small GTPases in 2D and 3D epithelial cell cultures. J Cell Biol.

[41] Green KN, Billings LM, Roozendaal B, McGaugh JL, LaFerla FM (2006). Glucocorticoids increase amyloid-beta and tau pathology in a mouse model of Alzheimer’s disease. J Neurosci.

[42] Jeong YH, Park CH, Yoo J, Shin KY, Ahn SM, Kim HS (2006). Chronic stress accelerates learning and memory impairments and increases amyloid deposition in APPV717I-CT100 transgenic mice, an Alzheimer’s disease model. FASEB J.

[43] Moore S, Evans LD, Andersson T, Portelius E, Smith J, Dias TB (2015). APP metabolism regulates tau proteostasis in human cerebral cortex neurons. Cell Rep.

[44] Sun J, Roy S (2018). The physical approximation of APP and BACE-1: a key event in alzheimer’s disease pathogenesis. Dev Neurobiol.

[45] Nixon RA (2005). Endosome function and dysfunction in Alzheimer’s disease and other neurodegenerative diseases. Neurobiol Aging.

[46] Small SA (2008). Retromer sorting: a pathogenic pathway in late-onset Alzheimer disease. Arch Neurol.

[47] Bhalla A, Vetanovetz CP, Morel E, Chamoun Z, Di Paolo G, Small SA (2012). The location and trafficking routes of the neuronal retromer and its role in amyloid precursor protein transport. Neurobiol Dis.

[48] Sannerud R, Declerck I, Peric A, Raemaekers T, Menendez G, Zhou L (2011). ADP ribosylation factor 6 (ARF6) controls amyloid precursor protein (APP) processing by mediating the endosomal sorting of BACE1. Proc Natl Acad Sci USA.

[49] Sheehan P, Waites CL (2017). Coordination of synaptic vesicle trafficking and turnover by the Rab35 signaling network. Small Gtpases.

[50] Peng KY, Perez-Gonzalez R, Alldred MJ, Goulbourne CN, Morales-Corraliza J, Saito M (2019). Apolipoprotein E4 genotype compromises brain exosome production. Brain.

[51] Huang YA, Zhou B, Wernig M, Sudhof TC (2017). ApoE2, ApoE3, and ApoE4 differentially stimulate APP transcription and Abeta secretion. Cell.

[52] Goetzl EJ, Ledreux A, Granholm AC, Elahi FM, Goetzl L, Hiramoto J (2019). Neuron-derived exosome proteins may contribute to progression from repetitive mild traumatic brain injuries to chronic traumatic encephalopathy. Front Neurosci.

