# Supplementary material for: Rab35 and glucocorticoids regulate APP and BACE1 trafficking to modulate Aβ production
Source: Cell Death Dis. 2021 Dec 8;12(12):1137. doi: 10.1038/s41419-021-04433-w (PMC8651661; doi:10.1038/s41419-021-04433-w)
Supplement: Supplementary file 1 — Supplemental Figure Legends [file 41419_2021_4433_MOESM1_ESM.docx]

**Supplemental Figure Legends**

**Figure S1. Rab35 knockdown and effect on recycling endosome morphology**

A-B) Representative immunoblots and quantification of Rab35 knockdown in N2a cells expressing APP-GFP or FLAG-BACE, HA control, and control siRNA (siCtrl) or siRNA to knockdown Rab35 (siRab35). Blots were probed for Rab35 and tubulin, and values were normalized to tubulin and expressed as a fraction of siCtrl condition. Cells transfected with siRab35 show a ~55% reduction in Rab35 expression, regardless of whether they co-express APP-GFP or FLAG-BACE1 (**P=0.0059; unpaired two-tailed t-test, n=3 samples/condition). C-D) Representative immunoblots and quantification of Rab35 knockdown in N2a cells expressing mCh control or mCh with an shRNA to knockdown Rab35 (shRab35). Blots were probed for Rab35 and tubulin, and values were normalized to tubulin and expressed as a fraction of mCh control condition. Cells transduced with shRab35 show an ~50% reduction in Rab35 expression (**P=0.0028, unpaired two-tailed t-test, n=9-13 samples/condition). E) Quantification of Rab11 puncta size, showing that Rab35 overexpression does not alter Rab11 endosome morphology (n=56-68 cells/condition). All numeric data represent mean ± SEM.

**Figure S2. Rab35 does not promote degradation of APP, CTFs, or BACE1.**

A-C) Representative immunoblots and quantification of APP and APP CTF degradation in 14 DIV hippocampal neurons expressing GFP, GFP-Rab35 or shRab35. Neurons were treated with cycloheximide (CHX) for 0, 2, 4 or 8 hours, and probed for APP and tubulin. Values were normalized to tubulin and expressed as % of protein levels at 0h. Modulation of Rab35 levels does not alter APP and/or CTF degradation (n_APP_=7-8 samples per condition/time point, n_CTFs_=5-6 samples per condition/time point). D-E) Representative immunoblots and quantification of BACE1 degradation in 14 DIV hippocampal neurons expressing GFP, GFP-Rab35 or shRab35, treated with CHX as above and probed for BACE1 and tubulin. Modulation of Rab35 levels does not alter BACE1 degradation (n=3-4 samples per condition/time point).

**Figure S3. Rab35 does not affect APP retrograde trafficking.**

A) Schematic representation of APP retrograde trafficking assay, in which cell-surface APP was labeled with 22C11 antibody, cells were incubated for 0, 10, 30 or 60 minutes to allow for APP internalization, and finally, cells were immunostained with syntaxin-6 antibodies to label the TGN. B) Representative high-resolution images (Zeiss Airyscan) of APP retrograde trafficking time course in control cells, with cells outlined in gray, the TGN outlined in blue, and white arrowheads pointing to areas of colocalization in insets. APP colocalization with the TGN (insets) increases up to the 30 min chase timepoint and then decreases by 60 min. C) Schematic representation of BACE1 retrograde trafficking assay. Surface FLAG-BACE1 was labeled with FLAG antibody. D) Representative high-resolution images (Zeiss Airyscan) of BACE1 retrograde trafficking time course, showing BACE1 colocalization with the TGN (insets) increasing over time. E-F) Representative high-resolution images (Zeiss Airyscan) and quantification of APP retrograde trafficking in N2a cells expressing APP-GFP and HA vector control or HA-Rab35. Internalized APP (red) and syntaxin-6 (blue) are shown at 0 and 60 min time points post-labeling, with cells outlined in gray, the TGN outlined in blue, and white arrows pointing to areas of colocalization in insets. Overexpression of Rab35 does not significantly alter the colocalization of internalized APP with syntaxin-6 at any time point, indicating no change in retrograde trafficking (n=53-69 cells per condition/time point). G-H) Representative high-resolution images (Zeiss Airyscan) and quantification of N- and C-terminal APP colocalization, using 22C11 antibody to label the N-terminus (via cell-surface labeling) and GFP to mark the C-terminus in N2a cells expressing APP-GFP and HA control or HA-Rab35. 22C11 antibody (blue) colocalizes with GFP (green) approximately 50% of the time across timepoints and conditions (n=53-101 cells/condition, 4 experiments). White arrowheads in insets point to areas of colocalization. Scale bars: 5 µm; 1 µm for zoomed insets. All numeric data represent mean ± SEM.

**Figure S4. Rab35 differentially regulates APP and BACE1 localization to the TGN in hippocampal neurons.**

A-B) Representative immunoblots and quantification of WT and DN Rab35 expression in N2a cells expressing FLAG-BACE1 with HA control, HA-Rab35 WT, or HA-Rab35 DN. Immunoblots were probed for Rab35 and tubulin. Cells express WT Rab35 at higher levels than DN Rab35 (*P=0.0131, ***P=0.0009, ****P<0.0001; one-way ANOVA with Tukey’s multiple comparisons test, n=14-16 samples per condition). C-D) Representative immunoblots and quantification of OCRL knockdown in N2a cells expressing APP-GFP and either HA control or HA-Rab35, control siRNA (siCtrl) or siRNA against OCRL (siOCRL). Immunoblots were probed for OCRL and tubulin. Cells expressing siOCRL show a ~60% reduction in OCRL, regardless of whether they co-express HA control or HA-Rab35 (****P<0.0001; unpaired, two-tailed t-test, n=12 samples per condition). E-F) Representative images and quantification of APP colocalization with syntaxin-6 in 14 DIV hippocampal neurons expressing mCh, mCh-Rab35, or shRab35, with cells outlined in gray, the TGN area outlined in red, and white arrowheads in insets pointing to areas of colocalization. Rab35 overexpression or knockdown does not significantly alter APP colocalization with syntaxin-6 compared to the control condition (n=64-70 cells per condition, 3 independent cultures). G-H) Representative images and quantification of BACE1 colocalization with syntaxin-6 in hippocampal neurons expressing mCh, mCh-Rab35, or shRab35, with cells outlined in gray, the TGN area outlined in red, and white arrowheads in insets pointing to areas of colocalization. Overexpression of Rab35 increases BACE1 colocalization with syntaxin-6, while Rab35 knockdown does not significantly alter this value (**P=0.006; one-way ANOVA, Dunnet post-hoc analysis, n=66-69 cells/condition, 3 independent cultures). Scale bars: 10 µm; 1 µm for zoomed insets. I-J) Quantification of syntaxin-6 puncta density (I) and size (J) in hippocampal neurons expressing mCh, mCh-Rab35, or shRab35. Rab35 overexpression or knockdown does not affect either value (n=56-69 cells/condition). All numeric data represent mean ± SEM.

**Figure S5. Rab35 mediates internalization but not endocytic recycling of BACE1.**

A) Schematic representation of APP recycling assay, in which APP internalization and recycling were assessed by labeling cell-surface APP with 22C11 antibody followed by cell incubation for 0, 10, 30, or 60 minutes, and fixation and immunostaining with secondary antibodies to detect recycled or internalized APP. B) Representative images of APP recycling assay time course in control cells. Cells are outlined in gray, and white arrows point to recycled 22C11 antibody at the cell surface. C) Schematic representation of BACE1 recycling assay, in which BACE1 internalization and recycling were assessed by labeling cell-surface BACE1 with FLAG antibody followed by cell incubation for 0, 10, 30, or 60 minutes, and fixation and immunostaining with secondary antibodies to detect recycled or internalized BACE1. D) Representative images of BACE1 recycling assay time course, with cells outlined in gray and white arrows pointing to recycled FLAG antibody around the cell surface. E-G) Representative images and quantification of BACE1 internalization and recycling in N2a cells expressing FLAG-BACE1 and either HA or HA-Rab35. Internalized and recycled APP are shown at 0 and 60 min time points post-labeling (E), and are expressed in graphs as ratios of total BACE1 at each time point (F-G). Rab35 overexpression increases BACE1 internalization at early time points, but has no effect on BACE1 recycling dynamics (***P=0.0004, *P=0.0494 (internalization); 2-way ANOVA with Sidak post hoc analysis, n=127-143 cells per condition/time point, 3 experiments). H-I) Representative images and quantification of steady-state cell-surface levels of APP in N2a cells transfected with APP-GFP and HA control or HA-Rab35. Rab35 increases cell-surface APP, expressed as a ratio of cell surface to total protein (****P<0.0001; unpaired two-tailed t-test, n=54-64 cells/condition, 3 experiments). J-K) Representative images and quantification of steady-state cell-surface levels of BACE1 in N2a cells transfected with FLAG-BACE1 and HA control or HA-Rab35. Rab35 does not alter cell-surface BACE1 levels (n=46-70 cells/condition, 3 experiments). Scale bars: 5 µm. All numeric data represent mean ± SEM.

**Figure S6. Roles of OCRL and ACAP2 in APP endocytic recycling and BACE1 retrograde trafficking.** A-C) Representative images and quantification of APP internalization and recycling in N2a cells expressing APP-GFP and either HA or HA-Rab35, together with control siRNA (siCtrl) or siRNA to knockdown OCRL (siOCRL). B) Compared to vector control, overexpression of Rab35 increases APP internalization at the 60 min time point, and OCRL knockdown does not alter this effect (**P_HA+siCtrl vs. Rab35+siCtrl_=0.0069, ****P_HA+siCtrl vs. Rab35+siOCRL_<0.0001; 2-way ANOVA with Sidak’s post-hoc test, n=33-47 cells per condition/time point, 2 experiments. *Time* × *Condition* interaction F_6,460_=9.453, P<0.0001, overall *Condition* effect F_3,460_=8.431, P<0.0001). C) Compared to control, overexpression of Rab35 increases APP recycling at 60 min, and OCRL knockdown further increases this effect (*P_HA+siCtrl vs. Rab35+siCtrl_ =0.0152, ****P_HA+siCtrl vs. Rab35+siOCRL_< 0.0001, *P_Rab35+siCtrl vs. Rab35+siOCRL_=0.0400; 2-way ANOVA and Tukey’s multiple comparisons test, n=33-47 cells per condition/time point, experiments. *Time* × *Condition* interaction F_6,460_=4.537, P=0.0002, overall *Condition* effect F_3,460_=5.606, P=0.0009). D-E) Representative immunoblots and quantification of ACAP2 knockdown in N2a cells expressing FLAG-BACE1 and either HA control or HA-Rab35, with control siRNA (siCtrl) or siRNA against ACAP2 (siACAP2). Immunoblots were probed for ACAP2 and tubulin. Cells expressing siACAP2 show reduced levels of ACAP2, regardless of whether they co-express HA control or HA-Rab35 (**P=0.0078 by unpaired, two-tailed t-test, n=4 samples/condition). F-G) Representative high-resolution images (Zeiss Airyscan) and quantification of BACE1 retrograde trafficking in N2a cells expressing FLAG-BACE1 and either HA or HA-Rab35, with control siRNA (siCtrl) or siRNA to knockdown ACAP2 (siACAP2). Cells are outlined in gray and the TGN is outlined in blue, with white arrowheads pointing to areas of colocalization. Internalized BACE1 (red) and syntaxin-6 (blue) are shown at the 60 min time point post-labeling. Rab35 overexpression increases BACE1 colocalization with syntaxin-6 at 60 min, and ACAP2 knockdown does not alter this effect (*P_HA+siCtrl vs HA+siOCRL_=0.0215, **P_HA+siCtrl vs HA-Rab35+siCtrl_=0.0056; 2-way ANOVA with Tukey’s multiple comparison’s test, n=50-66 cells per condition/time point, 3 experiments. *Time* × *Condition* interaction F_6,702_=4.665, P=0.0001, overall *Condition* effect F_3,702_=4.417, P=0.0044). Scale bars: 5 µm; 1 µm for zoomed insets. All numeric data represent mean ± SEM.

**Figure S7. GCs do not impact BACE1 retrograde trafficking.**

A-B) Representative high-resolution images (Zeiss Airyscan) and quantification of BACE1 retrograde trafficking in N2a cells expressing FLAG-BACE1 and either HA or HA-Rab35, treated with GCs or vehicle control. Cells are outlined in gray, the TGN is outlined in blue, and white arrowheads in insets point to areas of colocalization. Internalized BACE1 (red) and syntaxin-6 (blue) are shown at the 0 and 60 min time points post-labeling. GC treatment does not alter BACE1 colocalization with the TGN at any time point (n=43-65 cells per condition/time point, 3 experiments). Scale bar: 5 µm; 1 µm for zoomed insets. All numeric data represent mean ± SEM.
