# Supplementary material for: Rab35 and glucocorticoids regulate APP and BACE1 trafficking to modulate Aβ production
Source: Cell Death Dis. 2021 Dec 8;12(12):1137. doi: 10.1038/s41419-021-04433-w (PMC8651661; doi:10.1038/s41419-021-04433-w)
Supplement: Supplementary file 2 — Supplemental Figures [file 41419_2021_4433_MOESM2_ESM.pdf]

Figure S1

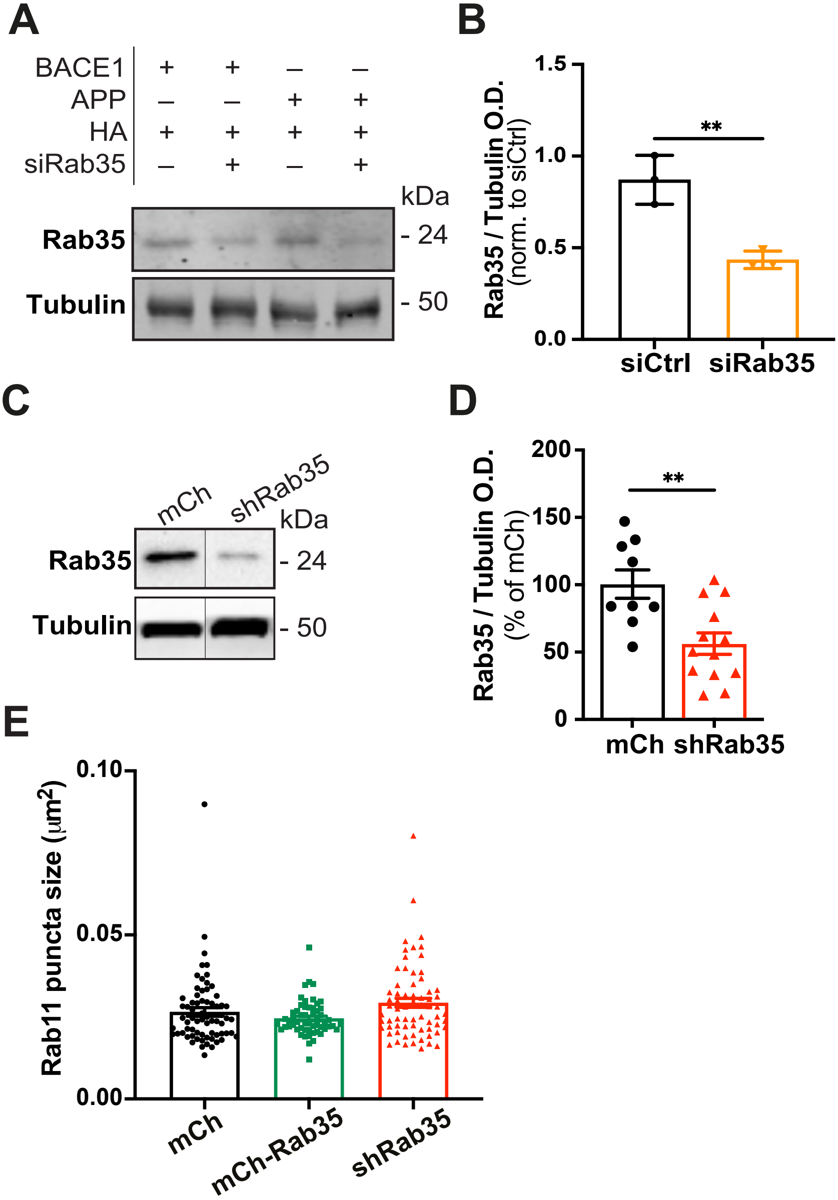
A
BACE1 + + – –
APP – – + +
HA + + + +
siRab35 – + – +
kDa
Rab35
- 24
Tubulin
- 50
B
Rab35 / Tubulin O.D. (norm. to siCtrl)
1.5
1.0
0.5
0.0
**
siCtrl
siRab35
C
mCh
shRab35
kDa
Rab35
- 24
Tubulin
- 50
D
Rab35 / Tubulin O.D. (% of mCh)
200
150
100
50
0
**
mCh
shRab35
E
Rab11 puncta size (μm²)
0.10
0.05
0.00
mCh
mCh-Rab35
shRab35

Figure S2

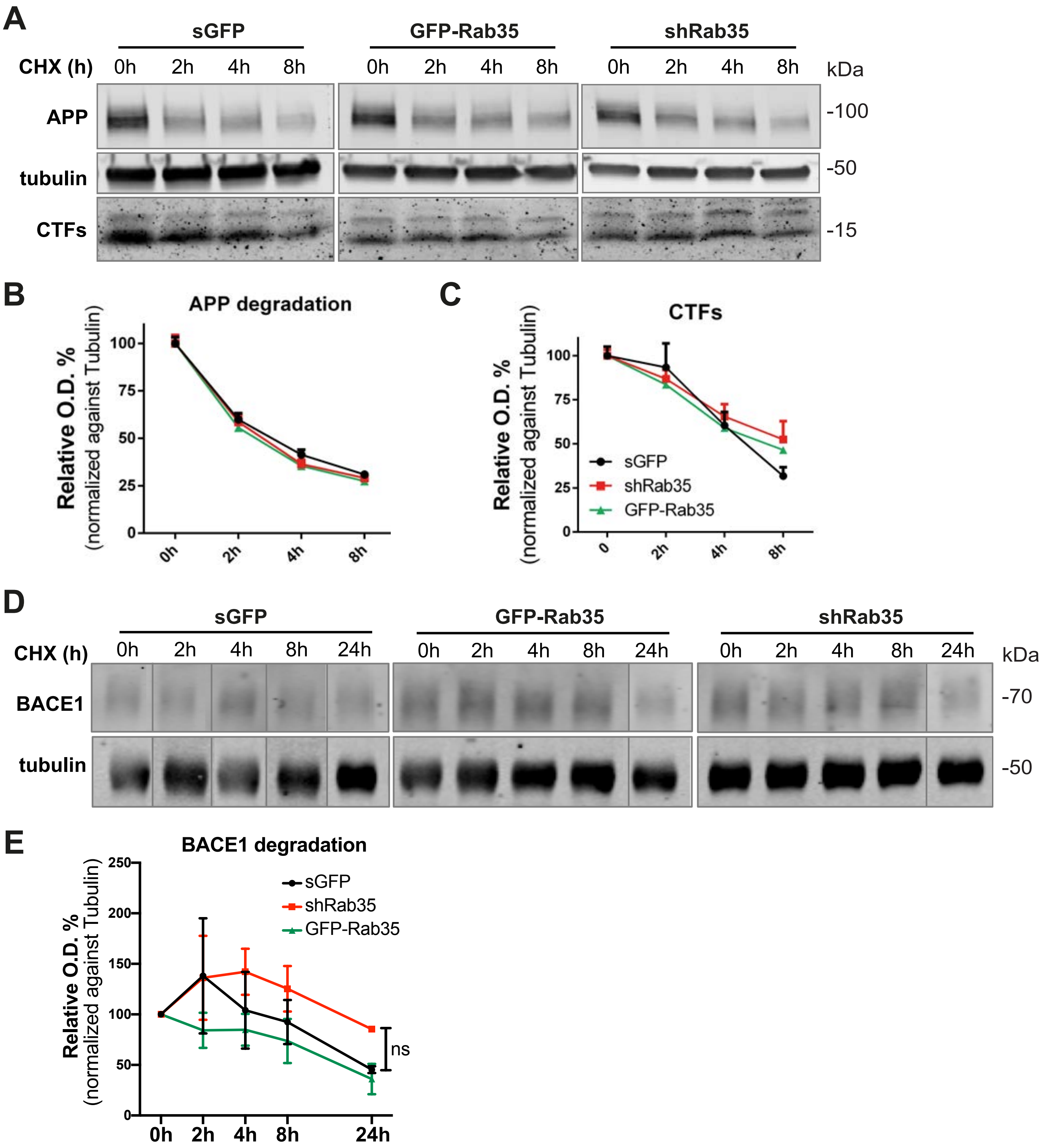
A
sGFP
GFP-Rab35
shRab35
CHX (h)
0h
2h
4h
8h
kDa
APP
tubulin
CTFs
-100
-50
-15
B
APP degradation
Relative O.D. %
(normalized against Tubulin)
100
75
50
25
0
0h
2h
4h
8h
C
CTFs
Relative O.D. %
(normalized against Tubulin)
sGFP
shRab35
GFP-Rab35
0
2h
4h
8h
D
sGFP
GFP-Rab35
shRab35
CHX (h)
0h
2h
4h
8h
24h
kDa
BACE1
tubulin
-70
-50
E
BACE1 degradation
Relative O.D. %
(normalized against Tubulin)
250
200
150
100
50
0
sGFP
shRab35
GFP-Rab35
ns
0h
2h
4h
8h
24h

Figure S3

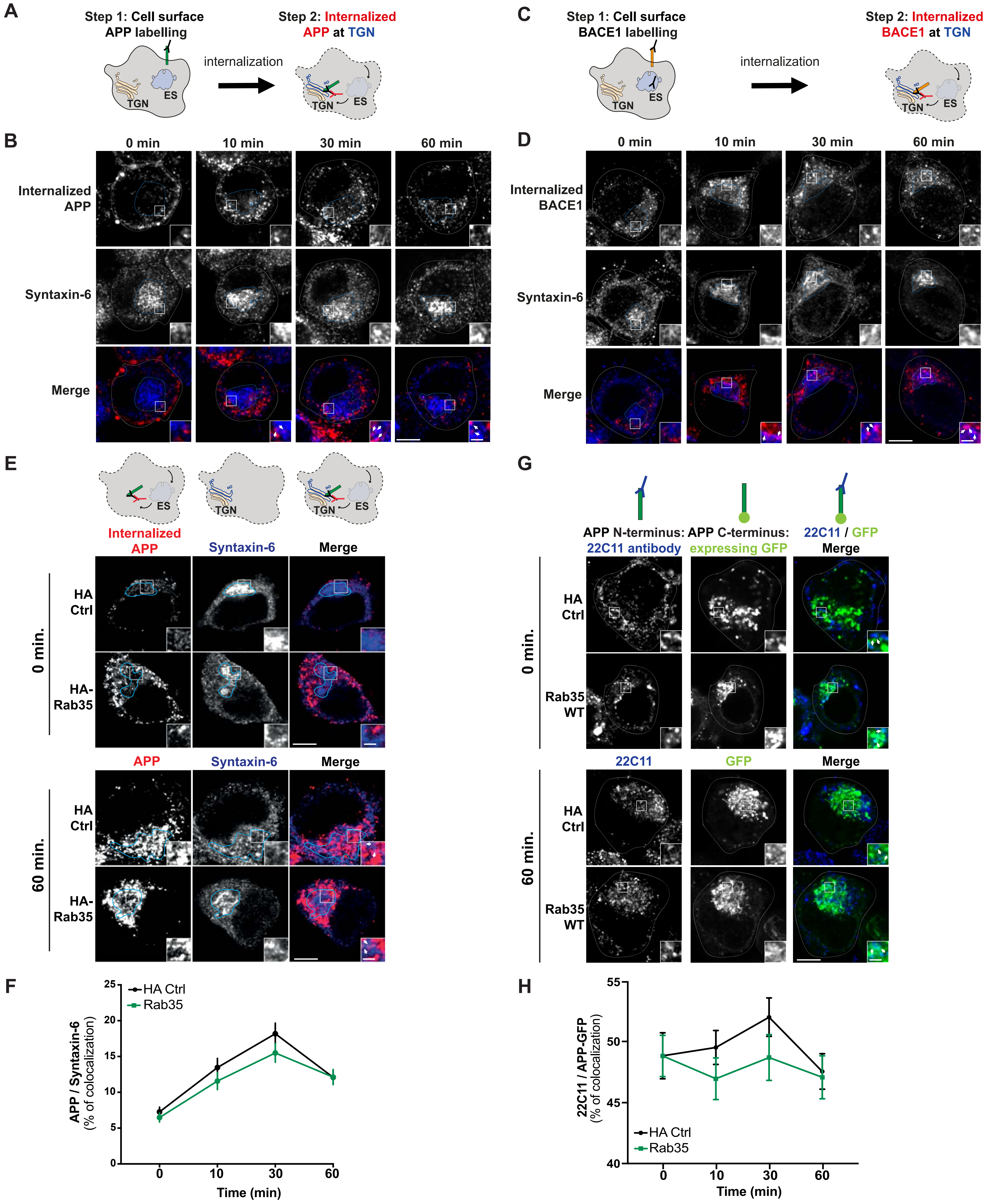

A
Step 1: Cell surface APP labelling
Step 2: Internalized APP at TGN
internalization
TGN
ES
C
Step 1: Cell surface BACE1 labelling
Step 2: Internalized BACE1 at TGN
B
0 min
10 min
30 min
60 min
Internalized APP
Syntaxin-6
Merge
D
Internalized BACE1
E
Internalized APP
APP
Syntaxin-6
Merge
HA Ctrl
HA-Rab35
0 min.
60 min.
G
APP N-terminus: 22C11 antibody
APP C-terminus: expressing GFP
22C11 / GFP Merge
22C11
GFP
Rab35 WT
F
APP / Syntaxin-6 (% of colocalization)
Time (min)
HA Ctrl
Rab35
H
22C11 / APP-GFP (% of colocalization)

Figure S4

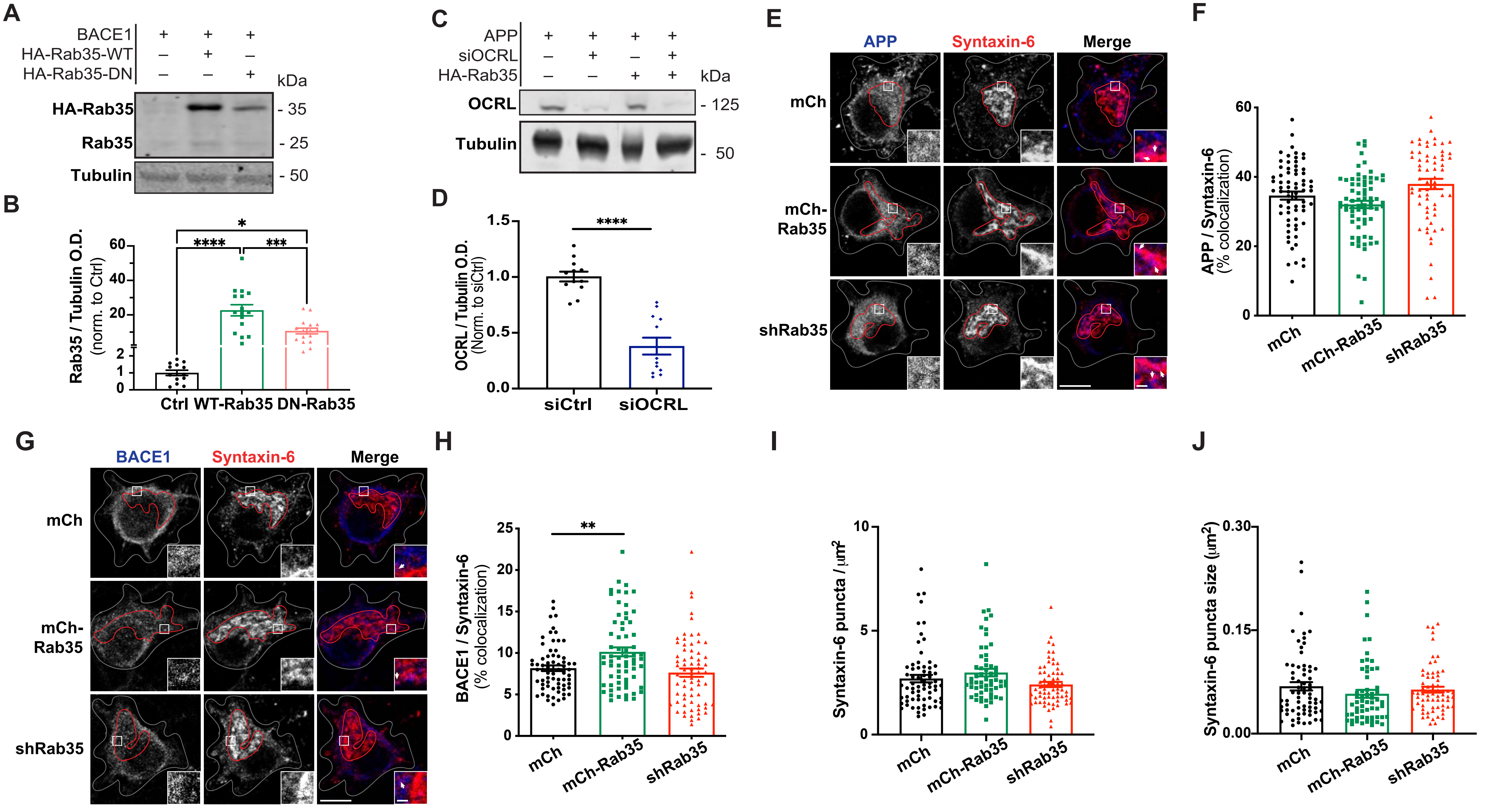
A
BACE1
HA-Rab35-WT
HA-Rab35-DN
kDa
HA-Rab35
Rab35
Tubulin
- 35
- 25
- 50
B
Rab35 / Tubulin O.D.
(norm. to Ctrl)
Ctrl
WT-Rab35
DN-Rab35
C
APP
siOCRL
HA-Rab35
kDa
OCRL
Tubulin
- 125
- 50
D
OCRL / Tubulin O.D.
(Norm. to siCtrl)
siCtrl
siOCRL
E
APP
Syntaxin-6
Merge
mCh
mCh-Rab35
shRab35
F
APP / Syntaxin-6
(% colocalization)
mCh
mCh-Rab35
shRab35
G
BACE1
Syntaxin-6
Merge
mCh
mCh-Rab35
shRab35
H
BACE1 / Syntaxin-6
(% colocalization)
mCh
mCh-Rab35
shRab35
I
Syntaxin-6 puncta / μm²
mCh
mCh-Rab35
shRab35
J
Syntaxin-6 puncta size (μm²)
mCh
mCh-Rab35
shRab35

Figure S5

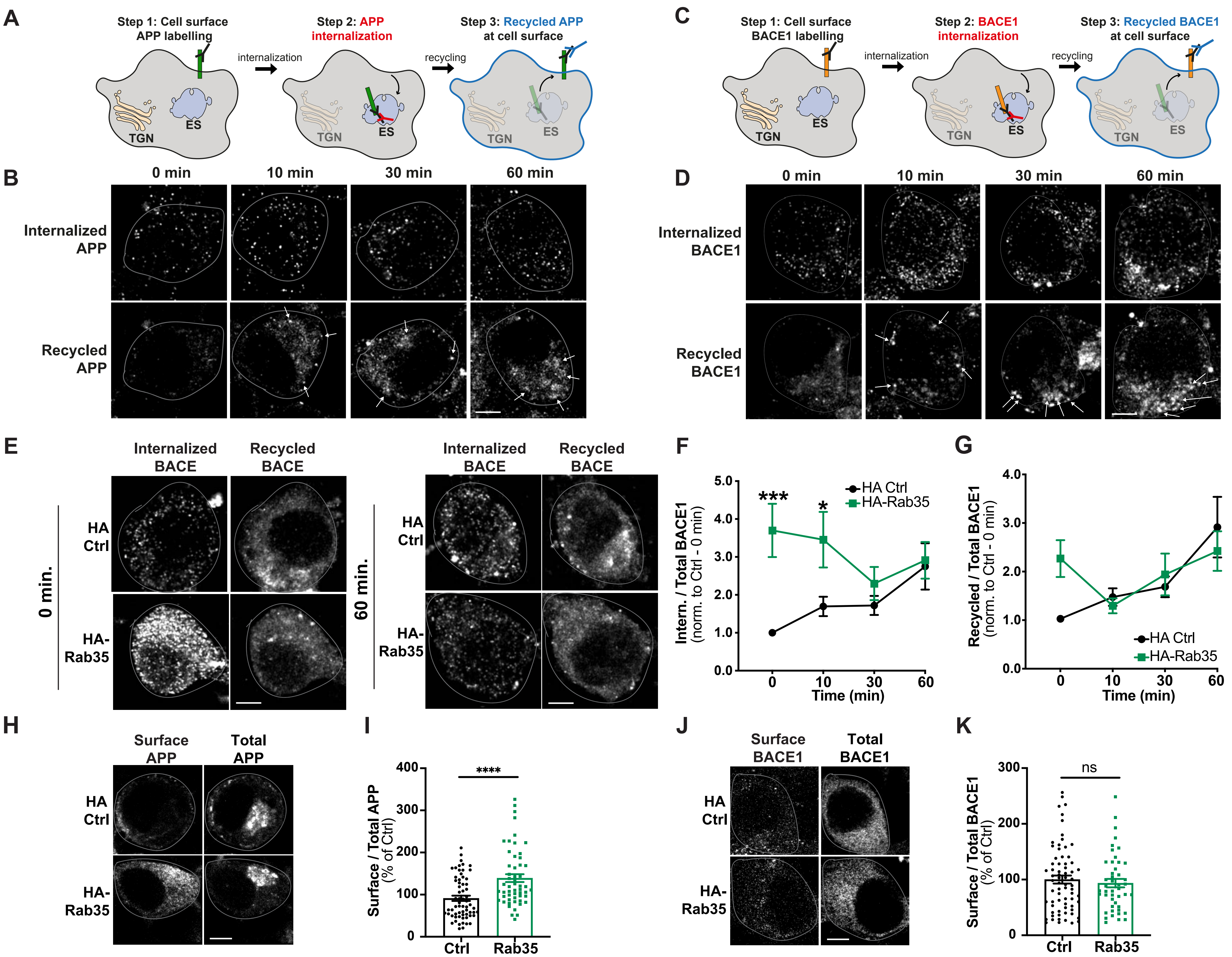

A
Step 1: Cell surface APP labelling
internalization
Step 2: APP internalization
recycling
Step 3: Recycled APP at cell surface
TGN
ES
B
0 min
10 min
30 min
60 min
Internalized APP
Recycled APP
C
Step 1: Cell surface BACE1 labelling
internalization
Step 2: BACE1 internalization
recycling
Step 3: Recycled BACE1 at cell surface
D
Internalized BACE1
Recycled BACE1
E
Internalized BACE
Recycled BACE
0 min.
60 min.
HA Ctrl
HA-Rab35
F
Intern. / Total BACE1 (norm. to Ctrl - 0 min)
HA Ctrl
HA-Rab35
***
*
Time (min)
G
Recycled / Total BACE1 (norm. to Ctrl - 0 min)
H
Surface APP
Total APP
I
Surface / Total APP (% of Ctrl)
****
Ctrl
Rab35
J
Surface BACE1
Total BACE1
K
Surface / Total BACE1 (% of Ctrl)
ns

Figure S6

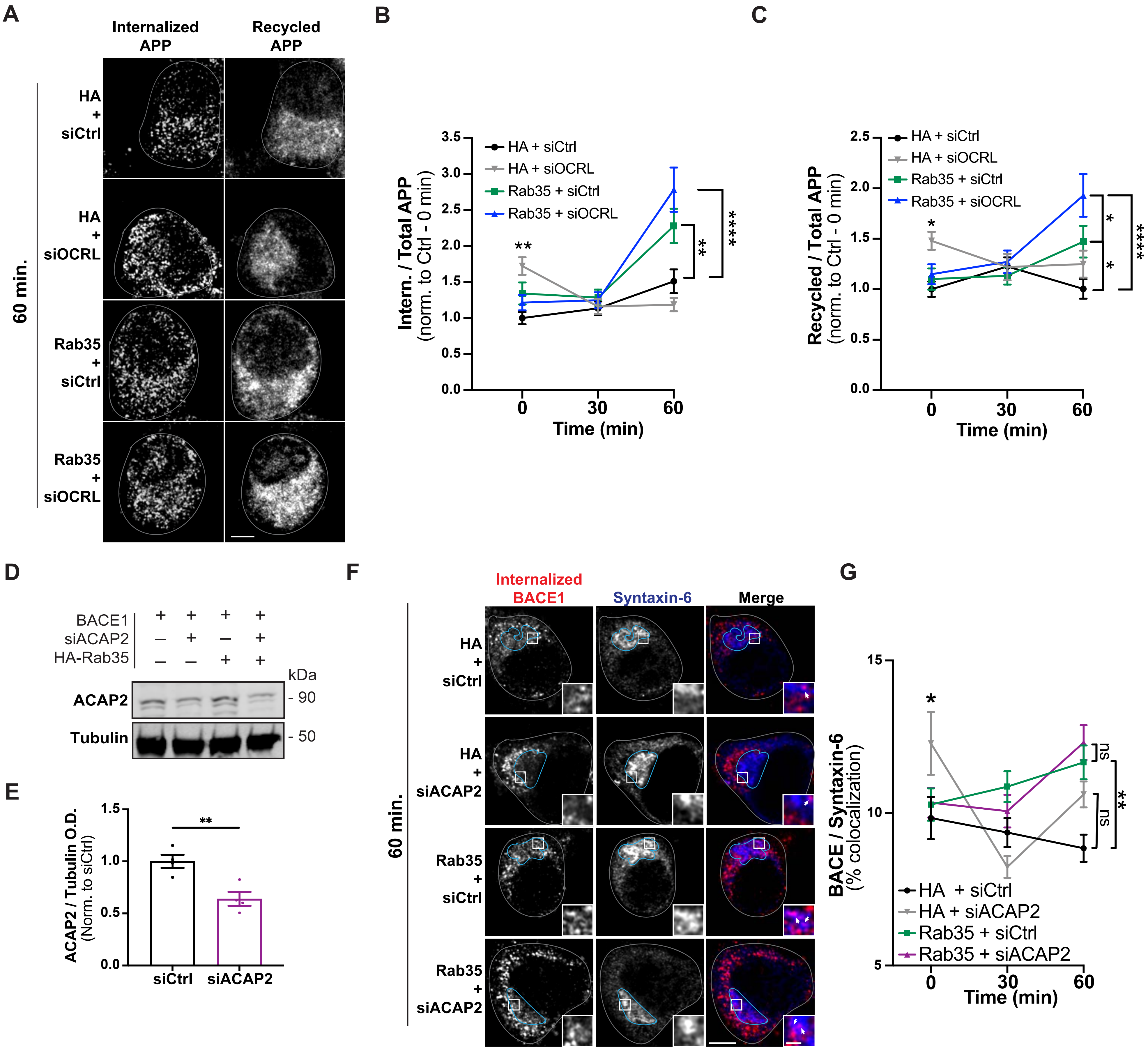

A
Internalized APP
Recycled APP
60 min.
HA + siCtrl
HA + siOCRL
Rab35 + siCtrl
Rab35 + siOCRL
B
Intern. / Total APP (norm. to Ctrl - 0 min)
Time (min)
HA + siCtrl
HA + siOCRL
Rab35 + siCtrl
Rab35 + siOCRL
C
Recycled / Total APP (norm. to Ctrl - 0 min)
Time (min)
D
BACE1
siACAP2
HA-Rab35
kDa
ACAP2
Tubulin
- 90
- 50
E
ACAP2 / Tubulin O.D. (Norm. to siCtrl)
siCtrl
siACAP2
F
Internalized BACE1
Syntaxin-6
Merge
HA + siCtrl
HA + siACAP2
Rab35 + siCtrl
Rab35 + siACAP2
G
BACE / Syntaxin-6 (% colocalization)
Time (min)
HA + siCtrl
HA + siACAP2
Rab35 + siCtrl
Rab35 + siACAP2
ns

Figure S7

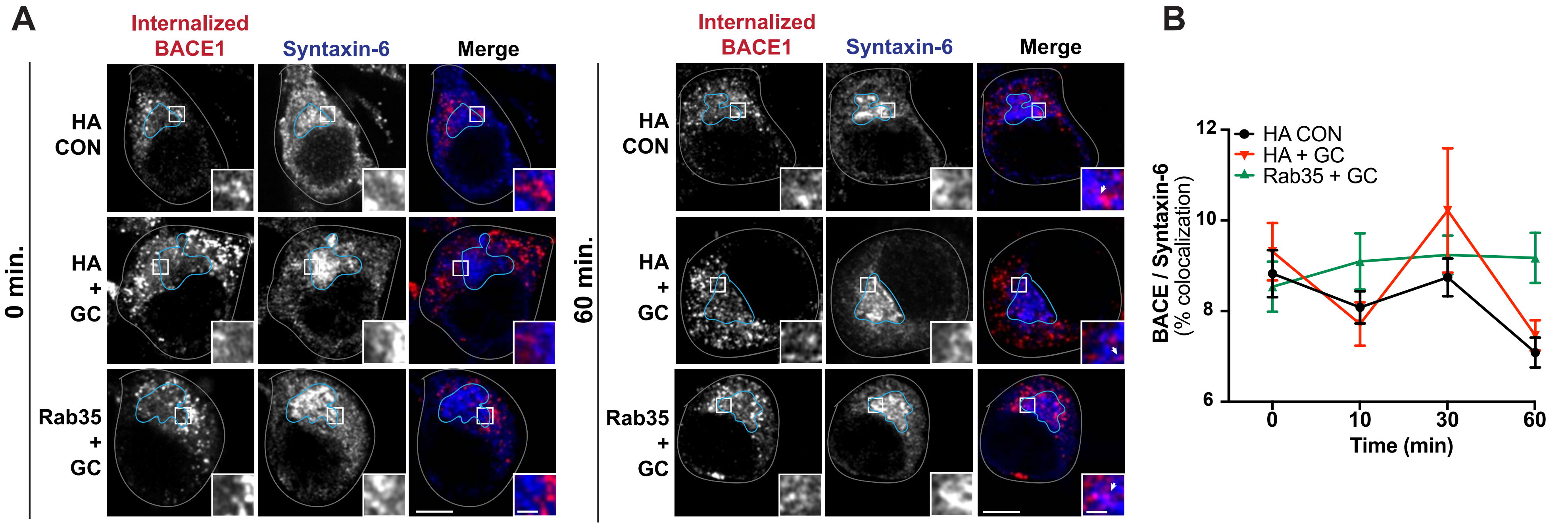
A
Internalized BACE1
Syntaxin-6
Merge
Internalized BACE1
Syntaxin-6
Merge
0 min.
60 min.
HA CON
HA + GC
Rab35 + GC
HA CON
HA + GC
Rab35 + GC
B
HA CON
HA + GC
Rab35 + GC
BACE / Syntaxin-6 (% colocalization)
12
10
8
6
0
10
30
60
Time (min)
